# Perceived AIGC saturation and AI content fatigue on short-video platforms: An appraisal-based structural equation modeling study

**DOI:** 10.1371/journal.pone.0360731

**Published:** 2026-10-16

**Authors:** Zedai Wang, Chao Tang

**Affiliations:** 1 School of Literature and Journalism, Hefei Normal University, Hefei, Anhui, China; 2 School of Animation, Jinling Institute of Technology, Nanjing, Jiangsu, China; Central China Normal University, CHINA

## Abstract

Artificial intelligence-generated content (AIGC) is increasingly embedded in short-video platforms, where algorithmic recommendation systems expose users to high volumes of rapidly produced synthetic media. Although prior research has examined generative AI from the perspectives of creativity, productivity, and technology adoption, less is known about how ordinary users evaluate repeated exposure to AI-generated short videos. This study develops and tests an appraisal-based model of AI content fatigue, distinguishing perceived AIGC saturation from users’ appraisals of low-quality prevalence, narrative homogenization, and content overload. A cross-sectional online survey was conducted among Chinese short-video users in May 2026. After excluding invalid responses, 513 valid questionnaires were analyzed using confirmatory factor analysis and structural equation modeling. The adjusted structural model showed acceptable fit to the data, χ²(1321) = 1991.541, χ²/df = 1.508, CFI = .946, TLI = .929, IFI = .947, and RMSEA = .031. Perceived AIGC saturation was positively associated with perceived low-quality prevalence, perceived narrative homogenization, and perceived content overload. Perceived low-quality prevalence had the largest estimated coefficient, followed by perceived content overload, whereas perceived narrative homogenization was not supported under bootstrap inference. AI content fatigue was negatively associated with satisfaction and continued viewing intention. Bootstrap analyses indicated selected path-specific indirect associations from perceived AIGC saturation to AI content fatigue, satisfaction, and continued viewing intention. Indirect pathways through perceived low-quality prevalence and perceived content overload were supported, whereas those through perceived narrative homogenization were not. The findings indicate that fatigue in AIGC-rich short-video environments is not simply a response to AI-generated content itself. It is more closely related to users’ appraisals of content quality, repetitiveness, and cognitive burden. The study contributes to social media fatigue research by conceptualizing AI content fatigue as a content-specific evaluative state and offers implications for platform governance, AIGC labeling, recommendation diversity, and quality-sensitive moderation.

## Introduction

Artificial intelligence-generated content (AIGC) is rapidly becoming part of everyday digital media experience. In creative industries, generative AI has been discussed as a tool that can improve productivity, accelerate ideation, and expand access to content production [1–3]. Recent empirical work also suggests that generative AI may enhance individual creative output while simultaneously reducing collective diversity in generated ideas or narratives [4,5]. This dichotomy is particularly pronounced in short-video ecosystems. While generative AI enables creators to scale production at unprecedented speed, algorithmic recommendation can amplify repeated formats, template-based narratives, and low-effort synthetic media. Consequently, the pressing question shifts from whether AIGC is technically capable to how audiences evaluate, filter, and become fatigued by repeated exposure to AI-generated content. For users, the key issue is not only whether AIGC is technically impressive, but whether repeated exposure to AI-generated short videos is associated with how they evaluate content quality, narrative variety, attentional burden, and willingness to continue watching.

Existing AIGC research has mainly examined production-side and adoption-side questions. Studies have investigated how creators, designers, students, and organizations adopt generative AI tools, often emphasizing perceived usefulness, innovation compatibility, autonomy, ethical fitness, or productivity gains [6–9]. Tang [7], for example, identified an autonomy paradox in China’s micro-short drama industry, showing that AIGC adoption may improve efficiency while raising concerns about creative control. Tang and Wang [8] further demonstrated that AIGC-enhanced learning analytics in film education requires ethical and pedagogical alignment rather than simple technological implementation. These studies provide an important foundation for understanding AIGC in Chinese creative and media contexts. However, they primarily focus on creators, educators, or institutional decision-makers. Less is known about ordinary users who encounter AIGC not as producers, but as audiences repeatedly exposed to AI-generated content in algorithmic short-video feeds.

This audience-side perspective is important because short-video platforms organize content consumption through continuous algorithmic recommendation, rapid switching, and high-frequency exposure. Unlike search-based media use, short-video users often encounter content passively through algorithmic feeds. As a result, AIGC may be experienced not as an isolated tool or novelty, but as a recurring feature of the platform environment. Users may perceive that AI-generated short videos are becoming saturated in their feeds, that low-quality AI content is increasingly prevalent, that narrative templates are repetitive, or that additional cognitive effort is required to identify and evaluate AI-generated videos. These perceptions may be associated with a form of fatigue that differs from general social media fatigue.

Social media fatigue has been widely studied as a psychological state associated with overload, exhaustion, reduced attention, and discontinuance intentions [10–13]. Prior research has shown that information overload, social overload, system feature overload, and flow disruption can predict exhaustion or discontinuance in digital environments [14–16]. Yet most social media fatigue research treats platform content as a general category. It rarely asks whether fatigue may emerge from a specific content-production regime, such as AI-generated content. The present study addresses this gap by conceptualizing AI content fatigue as a user’s cognitive and affective fatigue specifically associated with identifiable AI-generated short videos.

The central argument of this study is that perceived AIGC saturation is associated with users’ evaluations of AI-generated short videos. The model focuses on three appraisal dimensions: perceived low-quality prevalence (PLQ), perceived narrative homogenization (PNH), and perceived content overload (PCO). PLQ concerns the perceived prevalence of low-quality AI-generated videos, PNH concerns perceived repetition in narrative forms, and PCO concerns the perceived cognitive burden of identifying, filtering, and evaluating AI-generated videos. These constructs represent subjective appraisals rather than objective properties of platform content.

This study makes three contributions. First, it shifts AIGC research from a supply-side adoption perspective to a demand-side fatigue perspective. While prior work has examined whether creators, designers, or learners accept AIGC [7–9], this study asks how users experience AIGC as a recurring content environment in algorithmic short-video feeds. Second, it distinguishes perceived AIGC saturation from objective platform saturation. The study does not claim to measure the true proportion of AI-generated content on short-video platforms; instead, it examines perceived saturation as a psychologically meaningful exposure appraisal that may shape attention, evaluation, and behavioral intention. Third, it differentiates AI content fatigue from general short-video fatigue by theorizing fatigue as a content-specific response to identifiable AI-generated videos.

Methodologically, this study uses a cross-sectional SEM design with a theory-informed, context-specific questionnaire to conduct a preliminary empirical assessment of the appraisal-based pathways linking perceived AIGC saturation, AI content fatigue, satisfaction, and continued viewing intention. Although the cross-sectional design does not allow strong causal inference, it provides an appropriate first-stage empirical test of a theoretically specified model in an emerging research context where validated AIGC-specific user experience measures remain limited.

China’s short-video environment offers a theoretically valuable context for this inquiry. Chinese platforms such as Douyin, Kuaishou, WeChat Channels, Bilibili, and Xiaohongshu combine intensive algorithmic distribution with highly competitive content production ecosystems. Prior studies of AIGC in Chinese creative industries and higher education show that China is not merely a local case, but a useful site for observing how generative AI becomes embedded in creative workflows and media systems [7–9]. In this sense, Chinese short-video platforms serve as an advanced global laboratory for examining tensions that are increasingly relevant to algorithmic media ecosystems: content abundance, AI-assisted production, perceived repetition, and user fatigue.

Accordingly, this study addresses the following research questions:

RQ1: How do users’ perceptions of AIGC saturation relate to their appraisals of AI-generated short videos?

RQ2: How do perceived low-quality prevalence, perceived narrative homogenization, and perceived content overload relate to AI content fatigue?

RQ3: How is AI content fatigue associated with satisfaction and continued viewing intention?

RQ4: Do the three appraisal mechanisms and AI content fatigue explain indirect associations between perceived AIGC saturation and downstream user responses?

## Literature review and hypothesis development

### AIGC in creative and algorithmic media ecosystems

AIGC has transformed debates about creativity, production efficiency, and human-machine collaboration. Early discussions of AI in creative industries emphasized both the promise of automation and the need to understand how AI tools reshape creative labor, authorship, and artistic judgment [1,17]. Recent experimental research has added nuance to this debate. Noy and Zhang [3] found that generative AI can improve productivity in writing tasks. Doshi and Hauser [4], however, showed that generative AI can increase individual creativity while reducing collective diversity. Similarly, Meincke et al. [5] reported that ChatGPT decreased idea diversity in brainstorming. Together, these findings suggest that generative AI can expand individual capacity while also increasing the risk of homogenized outputs.

This tension is important for short-video platforms. Short-video production rewards speed, replicability, and attention capture. AIGC tools may lower production barriers and accelerate content generation, but these same affordances may also encourage repeated templates, visual shortcuts, and rapid imitation. In creator-side research, Tang [7] showed that AIGC adoption in China’s micro-short drama industry involves a paradox between efficiency and creative autonomy. Tang and Wang [8] further argued that AIGC integration in film education should be evaluated through ethical and creative-pedagogical fitness rather than efficiency alone. These studies imply that AIGC should not be assessed only by output volume or technological capability. It should also be examined through the meanings and evaluations attached to AI-generated outputs.

The present study extends this argument to the audience side. Users do not directly observe the entire AIGC production process. Instead, they encounter content fragments in algorithmic feeds and infer whether videos are AI-generated based on labels, creator disclosure, platform cues, or visible features. Algorithmic media research has shown that users develop beliefs and feelings about algorithmic systems even when they do not fully understand how those systems operate [18,19]. Users also experience algorithms through everyday interpretations of how platforms select, prioritize, and repeat content in their feeds [20]. Therefore, user responses to AIGC are likely shaped not only by actual content properties, but also by perceived AI origin, perceived algorithmic distribution, and repeated exposure.

### From objective AIGC growth to perceived AIGC saturation

A core premise of this study is that perceived AIGC saturation is distinct from objective AIGC prevalence. Objective prevalence would require platform-level content auditing, detection systems, or access to platform logs. By contrast, perceived AIGC saturation refers to users’ subjective sense that AI-generated short videos frequently appear in their feeds and occupy a noticeable share of their browsing experience. This distinction matters because media effects often depend on perceived exposure and interpretation, not only on objective exposure.

Algorithmic awareness research supports this emphasis on perception. Zarouali et al. [21] developed the Algorithmic Media Content Awareness scale to measure users’ awareness that media content may be selected or recommended by algorithms. Gran et al. [19] argued that algorithm awareness may create a new form of digital divide, because users differ in their ability to recognize and interpret algorithmic mediation. Dogruel et al. [22] similarly conceptualized algorithm literacy as a measurable competence among internet users. These studies suggest that users actively form beliefs about the systems and content they encounter, even when those beliefs are incomplete.

In the context of AIGC, perceived saturation may function as the first stage of user appraisal. When users repeatedly encounter content they identify as AI-generated, they may begin to treat AIGC as a salient category within the platform environment. However, perceived saturation alone does not determine whether users respond positively or negatively. Some users may enjoy AI-generated videos, while others may find them repetitive, low-quality, or cognitively demanding. Therefore, this study treats perceived AIGC saturation as an antecedent of more specific appraisals rather than as a direct explanation for fatigue.

H1a–c: Perceived AIGC saturation is positively associated with users’ appraisal mechanisms: (a) perceived low-quality prevalence, (b) perceived narrative homogenization, and (c) perceived content overload.

### Appraisal mechanisms and AI content fatigue

Drawing on the Stimulus–Organism–Response framework [23] and Cognitive Appraisal Theory [24], this study treats perceived AIGC saturation as an environmental stimulus associated with users’ evaluations of AI-generated short videos. Three appraisal mechanisms are examined: perceived low-quality prevalence (PLQ), perceived narrative homogenization (PNH), and perceived content overload (PCO).

PLQ and PNH represent content-focused appraisals. PLQ concerns users’ perceptions that low-quality AI-generated videos are common, whereas PNH concerns perceived repetition in story templates, plot structures, and character relationships. These appraisals reflect users’ subjective evaluations rather than objective properties of platform content. Previous research on AI disclosure suggests that perceived machine origin may influence evaluations of content credibility and source quality [25,26]. Generative AI use may also affect perceived authenticity [27], while users’ responses to algorithmic outputs may vary depending on whether those outputs are perceived as flawed or beneficial [28,29].

PCO represents a resource-oriented appraisal. It concerns the cognitive demands involved in identifying, filtering, and evaluating AI-generated videos in a rapidly changing feed. Unlike perceived saturation, which refers to how frequently AI-generated content appears, PCO concerns the effort required to process and assess such content. This distinction separates perceived processing demands from AI content fatigue itself.

Within the S–O–R framework [23], PLQ, PNH, and PCO represent organism-level appraisal processes through which users interpret an AIGC-rich media environment. AI content fatigue is modeled as a subsequent psychological state, whereas satisfaction and continued viewing intention represent downstream responses. The model was not intended to provide an exhaustive account of all possible appraisal processes. Perceived authenticity, deception, trust, novelty loss, and algorithmic intrusiveness were not measured in the present study and remain directions for future research.

*H2a–c: The three appraisal mechanisms are positively associated with AI content fatigue: (a) perceived low-quality prevalence, (b) perceived narrative homogenization, and (c) perceived content overload.*

### AI content fatigue, satisfaction, and continued viewing intention

AI content fatigue is conceptualized as a content-specific fatigue state involving reduced attention, cognitive tiredness, irritation, boredom, and diminished viewing interest when encountering AI-generated short videos. This construct draws from social media fatigue research but narrows the referent from the platform as a whole to a specific content category. Zhang et al. [13] developed a social media fatigue scale from cognitive, behavioral, and emotional perspectives, while Dhir et al. [11] linked social media fatigue with psychological well-being and compulsive use. These studies support the view that fatigue is not simply low satisfaction; it is a psychological state that may shape evaluation and behavioral intention.

In this study, AI content fatigue is expected to be negatively associated with satisfaction with AI-generated short-video experiences. Satisfaction reflects users’ overall evaluation of recent viewing experiences. If users experience AI-generated videos as tiring, irritating, or interest-depleting, they are likely to evaluate those experiences less favorably. This relationship is consistent with prior research linking overload and exhaustion to negative platform outcomes [12,14,15].

AI content fatigue is also expected to be negatively associated with continued viewing intention. Continued viewing intention refers to users’ willingness to keep watching AI-generated short videos in the future. Prior social media research has shown that fatigue and overload are associated with discontinuance or reduced use intentions [10,12,14]. In the AIGC context, users who feel tired of AI-generated short videos may not leave the platform entirely, but they may avoid, skip, or become less willing to watch AI-generated content.

H3a–b: AI content fatigue is negatively associated with (a) satisfaction with AI-generated short-video viewing and (b) continued viewing intention toward AI-generated short videos.

### Indirect effects of perceived AIGC saturation

The proposed model assumes that perceived AIGC saturation is associated with downstream responses through appraisal-based mechanisms and AI content fatigue. This is theoretically important because it avoids a simplistic claim that “more AI content causes fatigue.” Instead, perceived saturation is expected to matter because it increases the salience of AI-generated content and makes users more likely to form judgments about quality, similarity, and processing burden. This appraisal-based logic is consistent with overload and fatigue research, which shows that digital media outcomes depend not only on exposure, but also on how users interpret and experience information, system features, social demands, and attentional strain [12,14,15]. It is also consistent with algorithmic awareness research, which emphasizes that users’ beliefs about algorithmic mediation shape their affective and behavioral responses [18,19,21].

The model further expects indirect associations from perceived AIGC saturation to satisfaction and continued viewing intention through the three appraisal mechanisms and AI content fatigue. For example, users who perceive high AIGC saturation may also perceive more low-quality AI content; this appraisal may be associated with stronger fatigue, which may then be associated with lower satisfaction and weaker continued viewing intention. Similar indirect pathways may operate through perceived narrative homogenization and perceived content overload. Because the data are cross-sectional, these indirect effects should be interpreted as model-specified indirect associations rather than evidence of temporal or causal mediation.

H4a–c: Perceived AIGC saturation is indirectly associated with AI content fatigue through (a) perceived low-quality prevalence, (b) perceived narrative homogenization, and (c) perceived content overload.

H5a–c: *Perceived AIGC saturation is indirectly and negatively associated with satisfactio*n through sequential appraisal–fatigue pathways: (a) perceived AIGC saturation → perceived low-quality prevalence → AI content fatigue → satisfaction; (b) perceived AIGC saturation → perceived narrative homogenization → AI content fatigue → satisfaction; and (c) perceived AIGC saturation → perceived content overload → AI content fatigue → satisfaction.

H6a–c: Perceived AIGC saturation is indirectly and negatively associated with continued viewing intention through sequential appraisal–fatigue pathways: (a) perceived AIGC saturation → perceived low-quality prevalence → AI content fatigue → continued viewing intention; (b) perceived AIGC saturation → perceived narrative homogenization → AI content fatigue → continued viewing intention; and (c) perceived AIGC saturation → perceived content overload → AI content fatigue → continued viewing intention.

### Distinguishing AI content fatigue from general short-video fatigue

A final conceptual issue is the distinction between AI content fatigue and general short-video fatigue. General short-video fatigue may arise from prolonged use, entertainment overload, compulsive scrolling, or platform-level exhaustion. AI content fatigue, by contrast, refers specifically to fatigue associated with identifiable AI-generated short videos. This distinction is necessary because a user may be tired of the platform in general, tired of AI-generated content specifically, or both.

Prior research provides a strong basis for measuring general social media fatigue, but AIGC-specific fatigue requires a more precise referent [13]. Without this distinction, a study may mistakenly attribute general platform fatigue to AIGC exposure. Therefore, the present model includes general short-video fatigue as a related but separate construct. This allows the analysis to examine whether AI content fatigue retains explanatory value beyond general fatigue associated with short-video use.

This distinction also strengthens the study’s theoretical contribution. If AI content fatigue is empirically distinguishable from general short-video fatigue, it would suggest that AIGC changes not only production processes, but also audience-side fatigue mechanisms. Such a finding would extend social media fatigue research into the era of generative AI and provide a more precise vocabulary for platform governance. Rather than treating user fatigue as a generic consequence of “too much content,” the model identifies how perceived AI-generated content saturation, low-quality prevalence, narrative homogenization, and content overload may jointly shape user experience. To strictly isolate the specific fatigue associated with AIGC, this study incorporates general short-video fatigue, pre-existing AI attitudes, AI identification confidence, platform use intensity, and demographic variables as controls in the structural model testing.

The proposed model treats perceived AIGC saturation as an exposure appraisal, the three appraisal mechanisms as interpretive processes, AI content fatigue as a content-specific psychological state, and satisfaction and continued viewing intention as user responses. The full hypothesized cross-sectional SEM model is presented in Fig 1.

**Fig 1:**
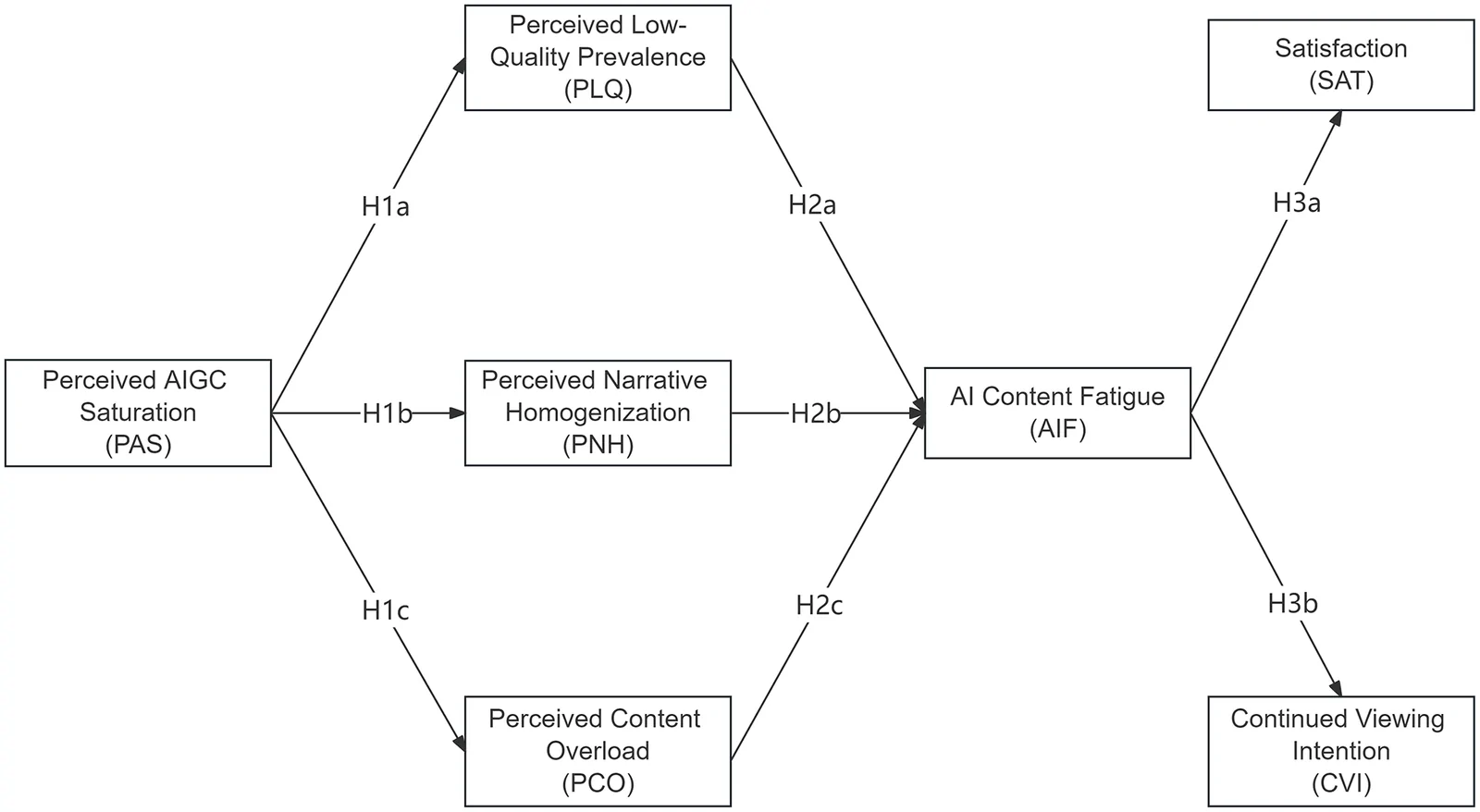
Conceptual model of perceived AIGC saturation, appraisal mechanisms, AI content fatigue, and user responses.

## Methods

### Research design and procedure

This study adopted a cross-sectional online survey design to test an appraisal-based SEM model of AI content fatigue in short-video environments. The design was appropriate for an initial empirical examination of an emerging phenomenon where AIGC-specific user experience constructs remain underdeveloped. The study combined theory-driven construct development with survey-based model testing, drawing on prior recommendations for scale development, preliminary measurement assessment, and structural equation modeling [30–33].

Questionnaire development and content review. The questionnaire was administered in Simplified Chinese. PAS, PLQ, and PNH were newly developed from their operational definitions and the characteristics of AI-generated short-video experiences. PCO items were adapted from the information-overload dimension of the Technology Overload Scale [34]. AIF items were adapted from the cognitive and emotional dimensions of the Social Media Fatigue Scale [13]. SAT and CVI items were adapted from satisfaction and continuance-intention measures based on the expectation-confirmation model [35].

For items adapted from English-language sources, the research team translated and contextually adapted the wording into Simplified Chinese. No independent back-translation or formal bilingual committee procedure was conducted.

Before formal data collection, five experts independently reviewed the candidate items in one review round. Their backgrounds covered media psychology, communication and human–computer interaction, psychometrics and statistics, survey methodology, and short-video industry or content governance. The review focused on item relevance, clarity, comprehensibility for Chinese short-video users, and contextual appropriateness. The research team consolidated the written feedback and revised the wording accordingly. No separate participant pilot study or formal cognitive interview was conducted.

PLQ8 was removed before formal administration because it assessed perceived informational value rather than the audiovisual, logical, and production-quality characteristics defined by PLQ. The final questionnaire therefore retained PLQ1–PLQ7. Other changes were wording refinements intended to clarify the construct boundaries of PLQ, PCO, and AIF. The decision was made during the expert-review stage before formal data collection and was not based on survey responses or statistical results. The complete item-source and retention information is provided in Supplementary Table S1 in S4 File.

Before formal data collection, the core measurement structure was specified a priori as seven correlated first-order factors: perceived AIGC saturation (PAS), perceived low-quality prevalence (PLQ), perceived narrative homogenization (PNH), perceived content overload (PCO), AI content fatigue (AIF), satisfaction (SAT), and continued viewing intention (CVI). This specification was based on the theoretical definitions of the constructs, the item-generation rationale, and the expert-review process. The present study did not include a separate exploratory factor analysis, pilot sample, or independent CFA sample. All 513 valid responses were used for the CFA and subsequent structural equation modeling. Accordingly, the CFA findings are interpreted as preliminary evidence regarding the proposed measurement structure rather than as definitive independent validation of a fully established scale.

Data were collected through an online questionnaire between 13 May 2026 and 19 May 2026. The questionnaire was distributed through Wenjuanxing, WeChat, Xiaohongshu, QQ, and related online networks. No monetary or material compensation was provided for participation. A total of 536 responses were collected. After excluding invalid responses according to the pre-specified data quality rules, 513 valid responses remained for analysis. Because all variables were measured in a single survey wave, the findings should be interpreted as cross-sectional associations rather than evidence of temporal or causal effects.

### Participants and sampling

Participants were adult short-video users in mainland China. To qualify for the study, participants had to be at least 18 years old, reside in mainland China, use short-video platforms at least three days per week during the previous 30 days, and report having encountered reasonably identifiable AI-generated short videos on their most frequently used platform. Participants who did not meet these screening criteria were directed out of the questionnaire before entering the main survey. The survey platform did not retain a separate analyzable count of respondents screened out before entering the core questionnaire.

The final analytic sample consisted of 513 valid respondents. No formal a priori sample-size or power calculation was conducted, and no target sample size was registered before data collection. The final sample size was determined by the feasible recruitment of eligible adult short-video users. The freely correlated seven-factor CFA contained 83 estimated parameters, whereas the covariate-adjusted structural model contained 449 estimated parameters. Accordingly, the sample of 513 respondents should be interpreted as a feasibility-based sample supporting preliminary measurement and structural estimation, rather than as a sample size justified by prospective power analysis. Participants reported their most frequently used short-video platform, AI-generated content exposure frequency, platform use intensity, AI identification confidence, and demographic information. The sample was obtained through online convenience and network-based recruitment rather than probability sampling. Therefore, the sample should not be interpreted as nationally representative of all Chinese short-video users. Instead, it provides a relevant sample of adult users who had recent exposure to identifiable AI-generated short videos.

### Measures

All core constructs were measured using seven-point Likert-type items ranging from 1 = strongly disagree to 7 = strongly agree. Items referred to participants’ experiences on their most frequently used short-video platform during the specified recall period.

AI-generated short videos were defined using an umbrella operational definition. Fully generated videos referred to videos in which major visual, audio, animation, character, script, or other audiovisual elements were generated by generative artificial intelligence. Substantially AI-assisted videos referred to videos in which generative artificial intelligence materially contributed to or transformed one or more major audiovisual or script elements. Both categories were included when the content could reasonably be identified as AI-related. Participants were instructed to rely on platform labels, creator disclosures, account information, or visible generative features. No standardized video examples were presented, and participants were not required to distinguish fully generated videos from substantially AI-assisted videos. Therefore, the measure captures perceived exposure to identifiable AI-related short videos rather than the objective prevalence of fully generated content.

PAS, PLQ, PNH, PCO, AIF, SAT, and CVI were measured using 4, 7, 4, 4, 6, 3, and 3 items, respectively. All items used a seven-point Likert-type response format and referred to participants’ experiences on their most frequently used short-video platform during the specified recall period. The complete item wording, coding, and item-source information are provided in Supplementary Table S1 in S4 File.

The model also included several covariates. General short-video fatigue was measured as fatigue associated with general short-video platform use, regardless of whether content was AI-generated. General short-video fatigue (GSVF) was measured using six context-adapted items referring to general short-video use, regardless of whether the viewed content was AI-generated. GSVF was modeled as a latent control construct in the adjusted structural model. The complete GSVF item wording and coding information are provided in Supplementary Table S5 in S4 File.

Negative AI attitude (AIATTneg) was computed as the mean of AIATT1–AIATT4, which assessed concern or unease about artificial intelligence. AIATT5, which assessed the positive potential of AI, was not included in the negative-attitude composite. Additional covariates included AI-identification confidence, AI-content exposure frequency, platform-use intensity, active following or searching of AI-content accounts, age, gender, education, and residential area type. The coding of these variables and the reference categories for categorical covariates are provided in the supplementary materials.

### Data quality screening

Several procedures were used to improve data quality. The hypotheses, measurement structure, eligibility criteria, and attention-check rules were defined before formal data collection and before inspecting the survey responses. These decisions were not registered in a formal preregistration protocol. The questionnaire included screening items, two instructed-response attention checks, and consistency checks related to short-video use and AI-generated content exposure. Responses were excluded if they failed the eligibility criteria or failed at least one of the two instructed-response attention checks. AC1 required the response “2 = Disagree,” and AC2 required the response “5 = Somewhat agree.” Completion time, long-string responding, contradictory responses, duplicate records, and missing core items were also examined as supplementary data-quality indicators. No additional responses were excluded on the basis of these supplementary checks. The complete coding and screening rules are provided in Supplementary S3 File.

The initial dataset contained 536 responses. After data screening, 513 valid responses were retained for analysis. Excluded responses were not used in the main SEM analysis.

### Analytical strategy

Data analysis proceeded in several steps. First, descriptive statistics were computed for all variables, including means, standard deviations, skewness, kurtosis, missingness, and response distributions. Data quality checks included attention check performance, response time, long-string responding, contradictory screening responses, and duplicate or suspicious entries.

Software, estimation, and data treatment. The CFA and SEM analyses were conducted using IBM SPSS Amos 26 with maximum likelihood estimation. The seven-point Likert items were treated as approximately continuous variables because they had seven response categories and showed limited skewness and kurtosis. Item-level skewness ranged from −.177 to  .188, and excess kurtosis ranged from −.425 to  .323. No missing core-item values were present in the final analytic sample, and no imputation was performed. The first loading of each latent factor was fixed to 1.0 for identification. No correlated indicator residuals were specified, and no post hoc structural paths or residual covariances were added on the basis of modification indices. Robust standard errors and scaled fit indices were not used in the AMOS maximum-likelihood analyses.

Second, the measurement model was evaluated before testing structural paths. The CFA and structural models were estimated as separate model specifications. The measurement model was a freely correlated seven-factor CFA comprising PAS, PLQ, PNH, PCO, AIF, SAT, and CVI, with no structural regression paths. The adjusted structural model retained the measurement component and added the hypothesized structural paths, GSVF, AIATTneg, exposure and usage controls, and demographic covariates. Fit indices were obtained separately from the corresponding AMOS output files. Confirmatory factor analysis was used to examine the factor structure of the latent constructs. Reliability, convergent validity, and discriminant validity were assessed using standardized factor loadings, Cronbach’s alpha, McDonald’s omega, composite reliability, average variance extracted, and HTMT ratios [36–38]. AI content fatigue and general short-video fatigue were modeled as related but distinct constructs to determine whether the AIGC-specific fatigue measure retained discriminant validity. Discriminant validity was assessed using both the latent-correlation matrix and the heterotrait–monotrait ratio of correlations (HTMT). Supplementary nested CFA comparisons further contrasted one-factor and two-factor specifications for PCO versus AIF and AIF versus GSVF. These supplementary CFA comparisons were estimated in R/lavaan using the same 513 valid responses. All 513 valid respondents were used for both the CFA and subsequent SEM analyses; therefore, the CFA did not constitute independent cross-sample validation.

Third, structural equation modeling was used to test the hypothesized paths. The model specified perceived AIGC saturation as the predictor, the three appraisal mechanisms as mediators, AI content fatigue as a content-specific fatigue state, and satisfaction and continued viewing intention as downstream responses. Indirect effects were evaluated using 5,000 usable bootstrap samples and bias-corrected 95% confidence intervals, a procedure recommended for evaluating indirect associations [39]. Because the data were cross-sectional, the bootstrap estimates were interpreted as model-specified indirect associations rather than evidence of causal mediation. The hypothesized indirect pathways were evaluated separately for perceived low-quality prevalence, perceived narrative homogenization, and perceived content overload. Specifically, H4a–H4c represented the indirect associations of PAS with AIF through PLQ, PNH, and PCO, respectively. H5a–H5c represented the sequential indirect associations of PAS with SAT through each appraisal mechanism and AIF. H6a–H6c represented the corresponding sequential indirect associations of PAS with CVI through each appraisal mechanism and AIF. The corresponding indirect-effect estimands were defined before model estimation. Because the data were cross-sectional, indirect effects were interpreted as model-specified indirect associations rather than evidence of causal mediation.

Two structural specifications were estimated. The unadjusted model included only the theoretically hypothesized paths among PAS, PLQ, PNH, PCO, AIF, SAT, and CVI. The covariate-adjusted model additionally included general short-video fatigue (GSVF), negative AI attitudes (AIATTneg), AI-identification confidence (CONF), AI-content exposure frequency (EXPOSURE), platform-use intensity (USE), active following of AI accounts (ACTIVE), and dummy-coded demographic variables.

Each covariate was specified as an exogenous predictor of AIF, SAT, and CVI. The reference categories were 18–24 years for age, high school/vocational school or below for education, township or rural area for residential area, and male for gender. Exogenous predictors were allowed to covary. The covariate-adjusted model was treated as the primary structural model, whereas the unadjusted model was retained for comparison.

The primary structural model was specified as an indirect-only model because the theoretical framework proposes that perceived AIGC saturation is associated with downstream outcomes through users’ appraisals and AI content fatigue. Thus, PAS was not assigned direct paths to AIF, SAT, or CVI in the primary model. This specification was used to test the proposed appraisal-based mechanism and was not intended to imply that direct associations were theoretically impossible. To examine whether this specification was overly restrictive, a sensitivity model was estimated by adding direct paths from PAS to AIF, SAT, and CVI while retaining all hypothesized paths and covariates.

For indirect effects, the bias-corrected 95% bootstrap confidence interval was treated as the primary inferential criterion. An indirect effect was considered supported only when its confidence interval did not include zero. Maximum-likelihood p values were reported as supplementary information and were not used to override the bootstrap-based conclusion. H4a–H4c represented the indirect associations of PAS with AIF through PLQ, PNH, and PCO, respectively. H5a–H5c represented the sequential indirect associations of PAS with SAT through each appraisal mechanism and AIF. H6a–H6c represented the corresponding sequential indirect associations of PAS with CVI through each appraisal mechanism and AIF. Because the data were cross-sectional, these estimates were interpreted as model-specified indirect associations rather than evidence of causal mediation [39]. Common-method bias was addressed through procedural and statistical approaches. Procedurally, the questionnaire used neutral wording, anonymity assurances, construct-specific instructions, separate construct blocks, and explicit temporal references to reduce evaluation apprehension and response consistency [40,41]. Attention-check items were used primarily to identify careless or inattentive responding. Statistically, Harman’s single-factor test was used as a supplementary diagnostic rather than as definitive evidence that common-method bias was absent. All core constructs were measured from the same 513 respondents in a single cross-sectional survey wave, using similarly structured seven-point Likert-type items. Several constructs also used evaluative or affective wording, which may increase the risk of shared method variance. Harman’s single-factor test was therefore treated only as a limited diagnostic and cannot establish that common-method variance had no substantive influence on the findings.

### Ethical considerations and open science

This study was based on an anonymous online questionnaire survey of adults. The study protocol was reviewed by the Academic Committee of Jinling Institute of Technology. Acting under the institutional procedures and policies for ethical review, the committee confirmed that the project met the criteria for exemption from full ethical review because it involved adult participants, anonymous data collection, no personally identifiable information, and minimal risk. The exemption statement was issued on May 13, 2026. No separate exemption or approval number was provided by the committee.

Before accessing the questionnaire items, participants read an online informed-consent statement describing the study purpose, voluntary participation, estimated completion time, potential risks, privacy protections, and the right to withdraw before submission. Consent was recorded electronically through a required consent item. Only participants who selected the consent option could proceed to the questionnaire; participants who did not consent were directed to the end of the survey. No handwritten signature or personally identifiable information was collected.

The survey recruited adult short-video users and did not collect personally identifying information such as names, phone numbers, short-video account IDs, precise locations, or contact details. The research team had access only to anonymous questionnaire responses. Data were stored in encrypted files accessible only to authorized members of the research team.

## Results

### Data screening and valid sample

A total of 536 questionnaire responses were collected through the online survey. Seven respondents failed AC1 only, and 16 respondents failed AC2 only. No respondent failed both attention checks. Therefore, 23 responses were excluded because they failed at least one attention check, leaving 513 valid responses for analysis. No additional responses were excluded because of missing core items, completion time, long-string responding, contradictory responses, or duplicate records. Among the 513 valid responses, the median completion time was 590 s, with an interquartile range of 516–673 s and a range of 60–904 s. Detailed screening rules and variable coding are provided in Supplementary S3 File. The final analytical sample therefore consisted of 513 valid responses. Detailed characteristics of the 513 valid respondents are reported in Supplementary Table S9 in S4 File. The table summarizes age, gender, education, residential area, most frequently used platform, AI-content exposure frequency, daily short-video use intensity, AI-identification confidence, and active following or searching for AI-content accounts. This sample was used for confirmatory factor analysis (CFA), structural equation modeling (SEM), and bootstrap tests of indirect associations.

Because the present study used a cross-sectional survey design, all paths reported below are interpreted as theoretically specified associations rather than causal effects. This interpretation is consistent with the study’s focus on perceived AIGC saturation, appraisal mechanisms, AI content fatigue, satisfaction, and continued viewing intention.

### Measurement model

The CFA and SEM analyses were conducted using the same 513 respondents. Therefore, the measurement results presented below should be interpreted as preliminary evidence for the proposed factor structure rather than as independent cross-sample validation. Before evaluating the measurement model, CMB was examined as a supplementary diagnostic check. A supplementary Harman’s single-factor test showed that the first unrotated factor accounted for 37.67% of the total variance. Although this value was below the conventional 50% reference point, Harman’s test has limited ability to detect common-method variance and cannot demonstrate that such variance had no material influence. The result was therefore interpreted only as a diagnostic indication, not as evidence that common-method bias was absent.

A freely correlated seven-factor CFA was conducted to evaluate the proposed measurement structure. The model included PAS, PLQ, PNH, PCO, AIF, SAT, and CVI and contained 83 estimated parameters and 413 degrees of freedom. The model fit the data well: χ²(413) = 461.760, χ²/df = 1.118, CFI = .995, TLI = .994, IFI = .995, RMSEA = .015, 90% CI [.001,  .022], RMR = .039, GFI = .946, AGFI = .935, and NFI = .951. These results provide preliminary evidence for the proposed seven-factor measurement structure. The reliability, factor-loading, and convergent-validity estimates are summarized in Table 1. The SRMR from the matched supplementary lavaan CFA specification was  .028.

**Table 1:** Measurement reliability, factor loadings, and convergent validity.

Construct	Items	Loading range	α	ω	CR	AVE
PAS	4	.737–.778	.860	.860	.851	.589
PLQ	7	.753–.791	.915	.915	.915	.607
PNH	4	.768–.795	.864	.864	.864	.614
PCO	4	.735–.812	.862	.862	.862	.610
AIF	6	.731–.775	.899	.899	.887	.567
SAT	3	.760–.781	.822	.822	.817	.599
CVI	3	.727–.773	.805	.805	.799	.569
GSVF	6	.753–.783	.895	.895	.895	.586

α = Cronbach’s alpha; ω = McDonald’s omega; CR = composite reliability; AVE = average variance extracted. The ω coefficients were calculated from the 513-case cleaned dataset using the corresponding item sets.

All standardized factor loadings ranged from  .727 to  .812, exceeding the commonly used  .70 benchmark. Composite reliability values ranged from  .799 to  .915, and average variance extracted values ranged from  .567 to  .614. These results indicate satisfactory indicator reliability, internal consistency, and convergent validity [42].

Discriminant validity was assessed using the complete latent-correlation matrix and the heterotrait–monotrait ratio of correlations (HTMT). In the seven-factor CFA, latent correlations ranged from −.550 to  .879, with the largest correlation observed between SAT and CVI (r = .879). The latent correlation between PCO and AIF was  .612. In the supplementary model including GSVF, the latent correlations were  .742 for AIF–GSVF and  .431 for PCO–GSVF. The corresponding HTMT values were  .608,  .741, and  .429, respectively. All HTMT values were below  .90. The complete latent-correlation and HTMT matrices are provided in Supplementary Tables S2 and S3 in S4 File.

We also compared one-factor and two-factor models for the closely related constructs. For PCO and AIF, the one-factor model showed χ²(419) = 980.848, CFI = .937, RMSEA = .051, and SRMR = .045, whereas the two-factor model showed χ²(413) = 462.662, CFI = .994, RMSEA = .015, and SRMR = .028. The two-factor model fit significantly better, Δχ²(6) = 518.186, p < .001, with ΔCFI = .057. For AIF and GSVF, the one-factor model showed χ²(608) = 1185.692, CFI = .947, RMSEA = .043, and SRMR = .045, whereas the two-factor model showed χ²(601) = 682.119, CFI = .993, RMSEA = .016, and SRMR = .028. The two-factor model again fit significantly better, Δχ²(7) = 503.573, p < .001, with ΔCFI = .046. These findings support the distinction between PCO, AIF, and GSVF. Nevertheless, some PCO and AIF items contain semantically related references to attention and information processing. The reported discriminant-validity evidence therefore supports their empirical separability but does not imply that the two constructs are completely independent.

### Structural model

For comparison, the unadjusted structural model included only the hypothesized paths among PAS, PLQ, PNH, PCO, AIF, SAT, and CVI. This model showed χ²(426) = 1007.883, χ²/df = 2.366, CFI = .935, TLI = .929, IFI = .935, and RMSEA = .052. The complete path estimates for the unadjusted model are provided in Supplementary Table S8 in S4 File for comparison.

The covariate-adjusted structural model contained 449 estimated parameters and 1,321 degrees of freedom. It included the hypothesized paths, GSVF, AIATTneg, CONF, EXPOSURE, USE, ACTIVE, and dummy-coded demographic covariates. The model showed χ²(1321) = 1991.541, χ²/df = 1.508, CFI = .946, TLI = .929, IFI = .947, RMSEA = .031, 90% CI [.029,  .034], RMR = .098, standardized RMR = .0696, GFI = .887, and AGFI = .848.

In the adjusted model, PAS positively predicted PLQ (β = .603, ML p < .001, bootstrap p < .001), PNH (β = .536, ML p < .001, bootstrap p < .001), and PCO (β = .465, ML p < .001, bootstrap p < .001). PLQ positively predicted AIF (β = .383, ML p < .001, bootstrap p = .001), and PCO positively predicted AIF (β = .254, ML p < .001, bootstrap p < .001). The path from PNH to AIF was not supported (β = .036, ML p = .298, bootstrap p = .467, bias-corrected 95% CI [−.052,  .116]). Thus, H1a–H1c and H2a/H2c were supported, whereas H2b was not supported in the adjusted model.

AIF was negatively associated with SAT (β = −.575, ML p < .001, bootstrap p = .001) and CVI (β = −.629, ML p < .001, bootstrap p = .001), supporting H3a and H3b. Among the control variables, GSVF positively predicted AIF (β = .527, p < .001), but was not significantly associated with SAT (β = −.002, p = .974) or CVI (β = .057, p = .406). EXPOSURE was positively associated with SAT (β = .111, p = .011) and CVI (β = .118, p = .007). The complete coefficients for AIATTneg, CONF, USE, ACTIVE, and all demographic dummy variables are reported in Supplementary Table S6 in S4 File.

The R² values in the covariate-adjusted model were  .364 for PLQ,  .288 for PNH,  .216 for PCO,  .747 for AIF,  .351 for SAT, and  .374 for CVI. Fig 2 summarizes the adjusted structural model. Table 2 reports the adjusted structural paths, and Supplementary Table S6 in S4 File reports all covariate paths. Estimation precision was assessed using the 95% confidence intervals reported for all standardized structural paths and indirect associations; no retrospective achieved-power calculation was performed.

**Table 2:** Structural path estimates.

Hypothesis	Path	β	ML p	Bootstrap p	Result
H1a	PAS → PLQ	.603	< .001	< .001	Supported
H1b	PAS → PNH	.536	< .001	< .001	Supported
H1c	PAS → PCO	.465	< .001	< .001	Supported
H2a	PLQ → AIF	.383	< .001	.001	Supported
H2b	PNH → AIF	.036	.298	.467	Not supported
H2c	PCO → AIF	.254	< .001	< .001	Supported
H3a	AIF → SAT	−.575	< .001	.001	Supported
H3b	AIF → CVI	−.629	< .001	.001	Supported

β = standardized path coefficient. ML p refers to the maximum-likelihood significance test. Bootstrap p refers to the bias-corrected bootstrap significance test based on 5,000 usable bootstrap samples. The complete coefficients for GSVF, AIATTneg, CONF, EXPOSURE, USE, ACTIVE, and all demographic dummy variables are reported in Supplementary Table S6 in S4 File.

**Fig 2:**
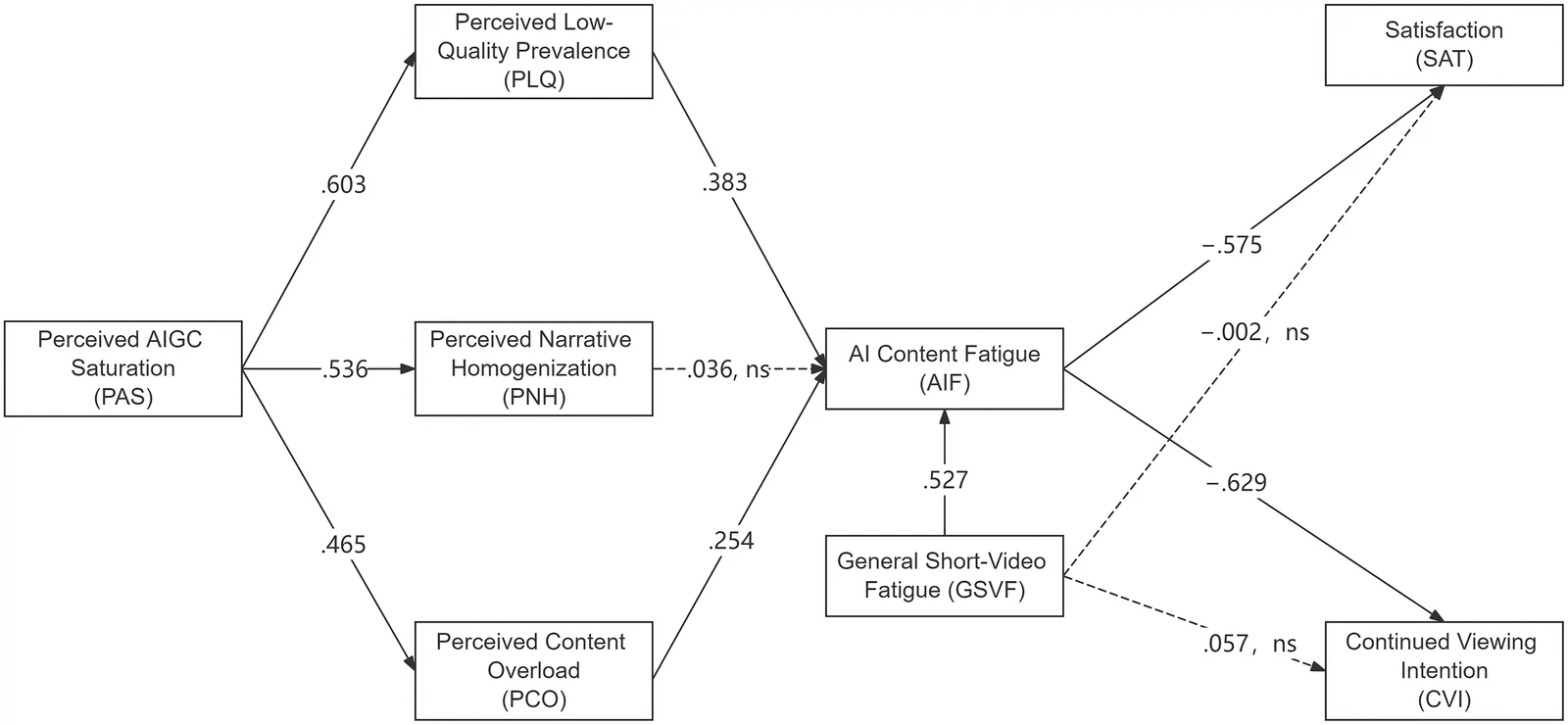
Standardized path coefficients in the covariate-adjusted structural model. Note. Values are standardized β coefficients rounded to three decimal places. Solid arrows indicate paths supported by the bias-corrected 95% bootstrap confidence interval, whereas dashed arrows indicate paths whose confidence interval included zero. PAS = perceived AIGC saturation; PLQ = perceived low-quality prevalence; PNH = perceived narrative homogenization; PCO = perceived content overload; AIF = AI content fatigue; SAT = satisfaction; CVI = continued viewing intention; GSVF = general short-video fatigue; ns = not supported. Only the focal structural paths and GSVF control paths are displayed. Other covariate paths were estimated but are omitted for visual clarity and reported in Supplementary Table S6 in S4 File.

### Explained variance

The covariate-adjusted model explained 36.4% of the variance in perceived low-quality prevalence, 28.8% of the variance in perceived narrative homogenization, and 21.6% of the variance in perceived content overload. The model explained 74.7% of the variance in AI content fatigue, 35.1% of the variance in satisfaction, and 37.4% of the variance in continued viewing intention. The corresponding R² values were  .364,  .288,  .216,  .747,  .351, and  .374, respectively, and are summarized in Table 3.

**Table 3:** Explained variance.

Endogenous construct	*R²*
Perceived low-quality prevalence (PLQ)	.364
Perceived narrative homogenization (PNH)	.288
Perceived content overload (PCO)	.216
AI content fatigue (AIF)	.747
Satisfaction (SAT)	.351
Continued viewing intention (CVI)	.374

### Indirect associations

Bias-corrected bootstrap analyses were conducted to examine the prespecified path-specific indirect associations. Bias-corrected 95% bootstrap confidence intervals were used as the primary inferential criterion. H4a, H4c, H5a, H5c, H6a, and H6c were supported because their confidence intervals excluded zero. H4b, H5b, and H6b were not supported because their confidence intervals included zero. Accordingly, H4–H6 received partial support rather than full support. The complete unstandardized estimates, standardized estimates, bootstrap standard errors, and bias-corrected 95% confidence intervals are reported in Table 4.

**Table 4:** Path-specific indirect effects in the covariate-adjusted model.

Hypothesis	Specific indirect path	Unstandardized estimate	Standardized estimate	Bootstrap SE	Bias-corrected 95% CI	Bootstrap p	Result
H4a	PAS → PLQ → AIF	.200	.231	.036	[.140, .275]	< .001	Supported
H4b	PAS → PNH → AIF	.017	.019	.024	[−.028, .066]	.447	Not supported
H4c	PAS → PCO → AIF	.102	.118	.022	[.064, .151]	< .001	Supported
H5a	PAS → PLQ → AIF → SAT	−.127	−.133	.032	[−.195, −.074]	.001	Supported
H5b	PAS → PNH → AIF → SAT	−.011	−.011	.016	[−.043, .019]	.466	Not supported
H5c	PAS → PCO → AIF → SAT	−.065	−.068	.016	[−.102, −.037]	< .001	Supported
H6a	PAS → PLQ → AIF → CVI	−.145	−.145	.033	[−.214, −.089]	.001	Supported
H6b	PAS → PNH → AIF → CVI	−.012	−.012	.018	[−.050, .020]	.461	Not supported
H6c	PAS → PCO → AIF → CVI	−.074	−.074	.018	[−.114, −.043]	< .001	Supported

Unstandardized estimates, bootstrap SEs, and bias-corrected confidence intervals were obtained from the AMOS bootstrap output. Standardized estimates were calculated from the products of the standardized component paths. A specific indirect effect was considered supported when its bias-corrected 95% confidence interval did not include zero.

### Direct-path sensitivity analysis

To assess whether the indirect-only specification was overly restrictive, we estimated a sensitivity model that added direct paths from PAS to AIF, SAT, and CVI. Both the sensitivity model and its comparison model were estimated in R/lavaan using the same 513 respondents, variables, estimator, and model parameterization.

The sensitivity model showed acceptable fit: χ²(1361) = 1790.419, CFI = .961, TLI = .957, IFI = .961, RMSEA = .0248, 90% CI [.0215,  .0279], and SRMR = .0689. Compared with the matched R/lavaan covariate-adjusted model without these direct paths, the addition of three direct paths produced a negligible change in fit, Δχ²(3) =  .937, p = .816. This Δχ² comparison was based solely on the two matched R/lavaan models and was not calculated from the AMOS fit statistics reported for the primary adjusted structural model.

None of the additional direct paths was statistically significant. The PAS → AIF path was β = −.005, p = .925, 95% CI [−.233,  .211]. The PAS → SAT path was β = −.057, p = .354, 95% CI [−.215,  .077]. The PAS → CVI path was β = −.042, p = .492, 95% CI [−.202,  .097]. These results were consistent with the indirect-only specification, although they do not constitute definitive proof of complete mediation.

## Discussion

### Summary of findings

These cross-sectional findings are consistent with an appraisal-based interpretation of AI content fatigue. Participants who reported stronger fatigue also tended to report perceiving AIGC-rich feeds as lower in value, more repetitive, or more cognitively demanding. The data do not establish that these appraisals produce fatigue, nor that fatigue necessarily follows the appraisals. The data do not establish whether participants accepted or rejected AIGC because of its AI origin. Within the constructs measured in this study, higher fatigue was more strongly associated with negative appraisals of perceived content quality and processing demands. Positive and neutral evaluations of AIGC were not measured.

Perceived low-quality prevalence had the largest estimated coefficient, followed by perceived content overload; perceived narrative homogenization was not supported in the adjusted model. This pattern is consistent with the possibility that fatigue is more closely related to perceived declines in content value and greater filtering and evaluation demands than to the mere presence of synthetic media. Because the data are cross-sectional, these associations cannot determine whether such appraisals preceded fatigue or were partly shaped by it. Perceived narrative homogenization showed a weaker association; this does not establish that formulaic content is incapable of producing fatigue.

Overall, perceived AIGC saturation was statistically associated with the appraisal measures rather than directly with fatigue in the primary indirect-only model. The findings are compatible with the proposed appraisal-based framework, but they do not establish that higher saturation precedes or leads to appraisals, or that appraisals precede fatigue.

### Theoretical contributions

This study contributes to AIGC and social media fatigue research by moving the analytical focus from production-side adoption to audience-side experience. Existing research has shown that generative AI can improve productivity, accelerate creative workflows, and reshape creative labor [1–3]. Related studies in Chinese creative and educational contexts have also shown that AIGC adoption involves tensions between efficiency, autonomy, ethics, and creative judgment [7,8]. The present study extends this line of work by examining what happens when users encounter AIGC not as creators or institutional adopters, but as audiences repeatedly exposed to AI-generated content in algorithmic short-video feeds.

The findings also refine the concept of social media fatigue. Prior studies have explained fatigue through information overload, communication overload, system feature overload, social overload, or prolonged platform use [10–15]. This study suggests that AI content fatigue is not reducible to general short-video fatigue or screen-time exhaustion. It is more closely associated with users’ appraisals of the perceived quality, density, and cognitive demands of AI-generated content. In this sense, AI content fatigue represents a content-specific fatigue state rooted in a particular mode of algorithmically circulated media production.

Perceived low-quality prevalence had the largest estimated standardized coefficient among the three appraisal paths. This finding extends overload-based fatigue research by showing that users’ fatigue may arise not only from the amount of information they encounter but also from perceived deterioration in content value. In AIGC-rich short-video environments, users may become tired when they repeatedly encounter videos perceived as visually awkward, narratively thin, technically compromised. In this sample, the observed associations were more closely related to participants’negative evaluations of content quality and processing demands than to the reported presence of AI-generated videos alone. Because positive and neutral appraisals were not measured, this pattern should not be interpreted as evidence that negative evaluations are the only relevant pathway.

Perceived content overload was another robust mechanism. This finding links AI content fatigue to the cognitive costs of navigating algorithmically amplified synthetic media. When users believe that AI-generated videos are widespread, they may need to engage in constant micro-evaluations: whether a video is AI-generated, whether it is worth watching, whether similar content should be skipped, and whether the platform is over-recommending such material. This process aligns with broader overload-based explanations of digital fatigue, while adding a content-specific layer rooted in AIGC recognition and appraisal [12,15].

In the covariate-adjusted model, perceived narrative homogenization showed a small and statistically unsupported association with AI content fatigue (β = .036, bootstrap p = .467, bias-corrected 95% CI [−.052,  .116]). This result should be interpreted cautiously. The estimated coefficient may reflect measurement overlap between narrative homogenization and other appraisal constructs, suppression or redistribution of shared variance among correlated appraisal variables and covariates, and sampling uncertainty. The finding does not establish that narrative homogenization is irrelevant or that its association with fatigue would be absent in other contexts. Future studies should use larger independent samples and improved measures that distinguish narrative similarity from perceived content quality and cognitive overload.

### Practical implications

The findings have implications for short-video platforms, content creators, and AIGC governance.

The findings suggest several hypotheses for future platform-design research, but the present study did not test any of these interventions. Platforms could experimentally compare binary AIGC labels with more granular disclosures, such as fully generated content, AI-assisted editing, AI-generated visuals with human scripting, and human-edited AIGC. Such designs could be evaluated for their effects on transparency, user understanding, and perceived processing burden [43]. However, the present study does not establish that more detailed labels would reduce fatigue or improve satisfaction.

Future experiments could also test user controls such as “show fewer AI-generated videos,” “reduce similar AI templates,” or “prioritize human-created content.” These proposals are consistent with human-centered AI principles emphasizing user control, reliability, and safety [44], but their effectiveness remains untested.

Perceived low-quality prevalence had the largest estimated standardized coefficient among the three appraisal paths to AI content fatigue. This descriptive comparison does not establish a statistically significant difference between the coefficients, but it suggests that perceived content quality may warrant attention in future platform-governance research. Automated detection of duplicate templates, repeated visual assets, unnatural body or facial rendering, low-effort synthetic narration, and excessive template reuse could be evaluated as possible inputs for quality-sensitive moderation. Such systems could also be examined in combination with user-feedback signals such as skipping, hiding, reporting, or selecting “not interested.”

Recommendation systems could likewise be evaluated in future studies using diversity-sensitive or fatigue-sensitive signals. Possible designs might test whether reducing near-duplicate templates, diversifying content sources, or temporarily lowering the density of AI-related videos affects perceived overload, fatigue, satisfaction, or viewing intention. These are tentative design proposals rather than interventions demonstrated to be effective by the present study.

For creators, the findings suggest that technical efficiency does not automatically translate into perceived value. AIGC may lower production costs and increase output volume, but excessive reliance on generic templates, awkward visual concealment, or repetitive narrative structures may undermine audience satisfaction. The practical challenge is not whether to use AI, but how to preserve narrative specificity, emotional authenticity, and visual credibility while using AI tools.

### Alternative explanations

Users may experience a form of algorithmic confounding, whereby the inherent repetition of recommender systems is misattributed to the generative nature of AI. Short-video platforms often expose users to similar topics, formats, and affective templates regardless of whether the content is human-made or AI-generated. Some users may therefore interpret algorithmic repetition as evidence of AIGC saturation, even when the underlying content is not fully AI-generated.

Pre-existing attitudes toward AI may also shape users’ appraisals. Users with stronger AI skepticism or anxiety may be more likely to notice technical flaws, assume content is AI-generated, or judge AI-assisted videos as lower in value. This possibility is consistent with research on algorithm aversion and user distrust of automated systems [28,45]. Future research should therefore examine whether pre-existing AI attitudes moderate the relationship between perceived AIGC saturation and AI content fatigue. Recent scale-development research has conceptualized AI fatigue as a multidimensional construct involving cognitive, emotional, behavioral, and physical components, providing a useful benchmark for future refinement of the present content-specific measure [46]. Related research on critical thinking in AI use has emphasized verification, epistemic motivation, and reflective judgment as components of critical engagement with AI outputs [47]. These dimensions may help future studies more precisely model the cognitive effort involved in identifying, filtering, and evaluating AIGC.

Users’ ability to identify AIGC is imperfect. Perceived AIGC saturation does not necessarily equal the objective proportion of AI-generated videos on a platform. The present study intentionally focuses on perceived saturation because user responses are shaped by subjective recognition and interpretation. Nevertheless, future studies should combine survey data with platform logs, content analysis, or experimental manipulation to distinguish perceived AIGC exposure from objectively measured AIGC exposure. Potential misclassification of fully generated and substantially AI-assisted content may have introduced measurement error. If this error was nondifferential, the observed associations may have been attenuated; if it was related to participants’ AI attitudes or identification confidence, the direction of bias is uncertain. Therefore, perceived AIGC saturation should not be interpreted as an objective estimate of platform-level AIGC prevalence.

Selective exposure may also influence the observed associations. Users who are already dissatisfied with short-video platforms may be more likely to notice low-quality AIGC, report higher fatigue, and express lower continued viewing intention. Although the structural model was theoretically specified, the cross-sectional design cannot rule out reverse or reciprocal associations.

### Limitations and future research

Several limitations should be acknowledged. First, the cross-sectional design limits causal and temporal interpretation. Although the model specified theoretically grounded paths from perceived AIGC saturation to appraisals, fatigue, satisfaction, and continued viewing intention, the data cannot establish temporal ordering or directionality. It is also possible that fatigue or dissatisfaction influenced participants’ negative evaluations of AI-generated content and their reports of perceived AIGC saturation. Longitudinal or experimental studies are needed to examine these reciprocal possibilities. Second, the measurement structure was assessed using a single sample. Although the seven-factor model was specified before data collection and showed acceptable CFA performance, the study did not include a separate exploratory-development sample or an independent CFA sample. Consequently, the factor loadings, fit indices, and reliability estimates may be sample-specific. Future research should replicate the measurement structure using an independent sample and, where feasible, follow a separate EFA–CFA sequence. The covariate-adjusted model was relatively parameter-intensive for the available sample. Although the reported estimates were obtained from a theoretically specified model, the complexity of the model may reduce replication stability. Future studies should use larger independent samples and prospective simulation-based sample-size planning to examine the stability of the measurement and structural estimates.

The study relies on self-reported perceptions of AIGC exposure. This is appropriate because the study focuses on perceived saturation, but it does not measure the objective amount of AI-generated content users encountered. Future research could combine survey responses with platform log data, computational content analysis, or expert-coded samples of short videos. In addition, all core variables were collected from the same respondents at the same time point and were measured using similarly formatted self-report scales. Several measures involved related evaluative or affective content. In particular, PCO and AIF items both referred to attention and information-processing experiences. This semantic proximity may have increased the covariance between the two constructs and may partly account for their observed association. Future research should develop and test wording that more sharply separates perceived processing demands from subsequent fatigue symptoms. Consequently, common-method variance cannot be ruled out, and the Harman test should not be interpreted as definitive evidence that this bias was absent. The model did not measure other potentially relevant appraisal processes, including perceived authenticity, deception, trust, novelty loss, or algorithmic intrusiveness. Therefore, the three mechanisms examined here should not be interpreted as an exhaustive set of explanations for AI content fatigue. Future studies should compare these alternative appraisals within a broader theoretical and measurement framework.

The sample was collected through an online survey and should not be interpreted as nationally representative. The appraisal measures in this study focused primarily on negative evaluations, including perceived low quality, narrative homogenization, and content overload. Positive or neutral evaluations, such as enjoyment, perceived novelty, usefulness, or creative appreciation, were not measured. These constructs may also share negative evaluative valence with AI content fatigue and dissatisfaction, potentially strengthening their observed associations. Therefore, the model cannot establish that users reject AIGC because of its AI origin or that negative appraisals are the only relevant determinants of fatigue and dissatisfaction. Future research should include balanced positive, neutral, and negative evaluations of AIGC. The findings are best understood as evidence from Chinese short-video users who were able to recognize or infer AI-generated content. Future studies should test the model across different platforms, countries, age groups, and media genres. The eligibility requirement that participants had encountered reasonably identifiable AI-generated short videos may have preferentially selected users with greater AI awareness or higher AI-content exposure. Users who encountered AI-related content but could not recognize or identify it may have been excluded before completing the core questionnaire. Therefore, the sample may overrepresent more AI-aware short-video users, and the findings should not be generalized to all short-video users. Future studies should recruit broader samples and combine self-reported exposure with objective platform or content-level measures.

The Chinese short-video context is theoretically valuable, but it also shapes the scope of interpretation. Chinese platforms represent a highly developed algorithmic media environment and a rapidly expanding AIGC production ecosystem [7,8]. Platform governance, labeling norms, user familiarity with AI, and creator incentives may differ across national contexts. Cross-cultural comparison would help determine whether the proposed appraisal mechanisms generalize beyond the present setting.

The relatively weak role of perceived narrative homogenization deserves further investigation. Future studies could examine whether narrative homogenization becomes more consequential under specific conditions, such as high exposure frequency, low genre involvement, strong AI skepticism, or repeated exposure to near-identical templates. Experimental designs could also manipulate narrative similarity and production cues separately to test whether users respond more strongly to sameness itself or to sameness combined with perceived low quality.

## Conclusion

This study examined the psychological mechanisms linking perceived AIGC saturation on short-video platforms to users’ downstream responses. Moving beyond the conventional focus on creator-side efficiency and institutional adoption [1,2,7,8], the findings support an appraisal-based framework of AI content fatigue. In the present sample, AI content fatigue was associated with negative appraisals of perceived low quality and cognitive burden. However, the cross-sectional design and the absence of positive or neutral appraisal measures prevent conclusions about whether users reject AIGC because of its AI origin or whether negative appraisals are the only relevant determinants of fatigue. Notably, perceived narrative homogenization played a more marginal role, suggesting that users in fast-paced media environments may tolerate formulaic narratives as long as they do not impose excessive cognitive strain or substantially compromise perceived content value.

These findings offer implications for the sociology of media and platform governance in the generative AI era. They dispel the deterministic notion that AIGC inherently degrades user satisfaction. Instead, they highlight the need for platforms to shift from binary “AI-generated” labeling toward more nuanced quality-control and user-control paradigms. These findings motivate future research to test whether recommendation diversity, duplicate-template suppression, quality-sensitive moderation, and user-control tools can reduce AI content fatigue; their effectiveness was not evaluated in the present study.

Several limitations warrant consideration. Given the cross-sectional nature of the survey data, the proposed paths represent theoretically specified associations rather than definitive causal trajectories. Perceived saturation also relies on self-reported appraisals, which may be influenced by users’ pre-existing AI attitudes, platform habits, or ability to identify AI-generated content. Future research should integrate longitudinal panel designs, computational content analysis, platform log data, or experiments to clarify the temporal dynamics between AIGC exposure, cognitive appraisals, fatigue, and viewing intentions. As generative technologies become more deeply embedded in everyday media consumption, sustained user engagement may depend less on the volume of AI content produced than on the quality, diversity, and cognitive manageability of algorithmic media environments.

## Supporting information

S1 File Questionnaire used in the online survey. S1 File Questionnaire.docx contains the full questionnaire administered to adult short-video users, including screening items, AI-identification items, construct measures, attention-check items, covariates, and demographic questions.
(DOCX)

S2 File Cleaned anonymized analysis dataset. S2 File Cleaned AnalysisDataset.csv contains the English-language, fully de-identified dataset with 513 valid responses retained after excluding 23 invalid responses from the 536 initial responses. The dataset contains no direct personal identifiers, sequential respondent identifiers, or retained free-text responses.
(CSV)

S3 File Codebook and data-screening rules. S3 File Codebook and data screening rules.xlsx provides variable names, item labels, construct mappings, coding rules, attention-check criteria, exclusion rules, valid-response identification, and scale-scoring procedures. The codebook contains no direct or indirect respondent identifiers.
(XLSX)

S4 File Supplementary tables S1–S10. S4 File Supplementary Tables S1-S10.docx contains the supplementary tables supporting the item-source and retention decisions, measurement-model results, latent correlations, HTMT analysis, one-factor versus two-factor CFA comparisons, GSVF measurement results, covariate path estimates, sensitivity analysis, unadjusted-model estimates, explained variance, participant screening, and sample characteristics.
(DOCX)
