# Supplementary material for: Perceived AIGC saturation and AI content fatigue on short-video platforms: An appraisal-based structural equation modeling study
Source: PLoS One. 2026 Oct 16;21(10):e0360731. doi: 10.1371/journal.pone.0360731 (PMC13674767; doi:10.1371/journal.pone.0360731)
Supplement: Supplementary file 1 [file pone.0360731.s001.docx]

**S1 File. Questionnaire used in the online survey**

Questionnaire on Content Experience and User Evaluation on Short-Video Platforms

*Documentation note. This file provides an English translation of the Simplified-Chinese questionnaire administered online. The participant-facing survey was presented in Simplified Chinese. No independent back-translation or formal bilingual committee procedure was conducted.*

**Introduction and informed consent**

Hello! We are conducting an academic study of users' experiences with short-video platforms. We aim to understand adults' exposure, evaluations, and recent feelings toward different types of content during their everyday use of short-video platforms.

This questionnaire takes approximately 10–12 minutes to complete. There are no right or wrong answers; please respond based on your actual experiences.

In this study, “AI-generated short videos” refers to short videos in which the visuals, characters, animations, voiceovers, text scripts, or other primary audiovisual content are generated by, or substantially assisted by, generative artificial intelligence. Please base your judgment primarily on platform labels, creator disclosures, account descriptions, or obvious generative features. Do not conclude that a video is AI-generated merely because it appears unpolished, repetitive, or unnatural.

This study poses minimal risk. Recalling repetitive, low-quality, or fatigue-inducing content may cause slight annoyance or discomfort. You may skip any question or exit the survey at any time.

The research team will not proactively collect your name, phone number, short-video account details, or precise location. Questionnaire data will be stored anonymously or in a de-identified manner and used solely for academic research. De-identified data, the questionnaire, data dictionaries, and analysis code may be shared on open-science platforms.

**CONSENT** Have you read and understood the information above, are you at least 18 years old, and do you voluntarily agree to participate?

Yes, I am at least 18 years old and voluntarily agree to participate.

No, I do not agree to participate, or I am under 18 years old. [Terminate survey]

**Part 1. Usage screening**

*Unless otherwise stated, the following screening questions refer to the past 30 days.*

**S1** Do you currently reside in mainland China?

Yes

No [Terminate survey]

**S2** Over the past 30 days, on average, how many days per week did you use short-video platforms?

0–2 days [Terminate survey]

3–4 days

5–6 days

Every day

**S3** Over the past 30 days, which short-video platform did you use most frequently? [Single choice]

Douyin (TikTok China)

Kuaishou

WeChat Channels

Bilibili

Xiaohongshu (RED)

**S4** Over the past 30 days, have you encountered reasonably identifiable AI-generated short videos on your most frequently used platform mentioned above?

Yes

Not sure [Terminate survey]

No [Terminate survey]

**Part 2. Exposure experience with AI-generated short videos in the past 30 days**

*The following items refer to your overall experience encountering AI-generated short videos on the selected platform over the past 30 days, rather than any specific video.*

*Please select the option that best reflects your actual feelings. Response scale: 1 = Strongly disagree; 2 = Disagree; 3 = Somewhat disagree; 4 = Neither agree nor disagree; 5 = Somewhat agree; 6 = Agree; 7 = Strongly agree.*

**PAS1** In the past 30 days, I have frequently seen AI-generated short videos in my algorithmic feed on this platform.

1 2 3 4 5 6 7

**PAS2** AI-generated short videos account for a noticeable proportion of the content I browse.

1 2 3 4 5 6 7

**PAS3** Even without actively searching for them, I frequently encounter AI-generated short videos.

1 2 3 4 5 6 7

**PAS4** I am likely to encounter AI-generated short videos across different browsing sessions.

1 2 3 4 5 6 7

**PLQ1** Many of the AI-generated short videos I encounter have obvious audiovisual production flaws.

1 2 3 4 5 6 7

**PLQ2** Many of the AI-generated short videos I encounter lack coherence in plot or expressive logic.

1 2 3 4 5 6 7

**PLQ3** A significant number of AI-generated short videos give me an overall impression of being unpolished.

1 2 3 4 5 6 7

**PLQ4** A significant number of AI-generated short videos lack necessary detailed refinement.

1 2 3 4 5 6 7

**PLQ5** Overall, low-quality AI-generated short videos are not uncommon in my encounters.

1 2 3 4 5 6 7

**PLQ6** I often notice unnatural details in faces, hands, or limbs within AI-generated short videos.

1 2 3 4 5 6 7

**PLQ7** I often notice unnatural adjustments made in these AI-generated short videos to conceal generation defects, such as blurred faces, deliberate obscuring of faces, or distorted limbs.

1 2 3 4 5 6 7

**PNH1** Different AI-generated short videos frequently adopt similar story templates.

1 2 3 4 5 6 7

**PNH2** The ways plots advance in AI-generated short videos are often identical or highly similar.

1 2 3 4 5 6 7

**PNH3** Character settings or interpersonal relationships in AI-generated short videos are frequently repetitive.

1 2 3 4 5 6 7

**PNH4** Different AI-generated short videos tend to follow highly similar patterns in terms of narrative arcs (beginning, development, climax, and conclusion).

1 2 3 4 5 6 7

**PCO1** Filtering which AI-generated short videos are worth watching places a heavy burden on me.

1 2 3 4 5 6 7

**PCO2** Judging whether an AI-generated short video is worth continuing to watch consumes too much of my attention.

1 2 3 4 5 6 7

**PCO3** When faced with AI-generated short videos that require filtering and judgment, I sometimes find it difficult to process the relevant information promptly.

1 2 3 4 5 6 7

**PCO4** When browsing AI-generated short videos, the information I need to process sometimes exceeds my available attention.

1 2 3 4 5 6 7

**AC1_Check** This is a quality-control item to ensure that you are reading carefully; please select “Disagree”.

1 2 3 4 5 6 7

**Part 3. Short-video use and AI-content identification**

*Unless otherwise stated, the following questions refer to the past 30 days.*

**IDB** What were your primary bases for determining whether a short video was related to AI? [Multiple choice; select all that apply]

IDB1 Platform labels such as “AI-generated” or similar labels.

IDB2 Disclosures made by the creator in the title, description, video, or comments.

IDB3 An account profile explicitly stating that it primarily publishes AI content.

IDB4 Shares or explanations by other people.

IDB5 Personal judgment based on visual, auditory, motion, or linguistic characteristics.

*Each IDB option is coded as an independent 0/1 indicator in the dataset.*

**LOWQ** Would you judge a video as likely being AI-generated solely because of its low quality or repetitive content?

Never

Rarely

Sometimes

Often

Always

**CONF** Overall, how confident are you in your ability to identify AI-generated short videos?

1 = Completely unconfident

2 = Rarely confident

3 = Somewhat unconfident

4 = Neutral

5 = Somewhat confident

6 = Confident

7 = Very confident

**USE** Over the past 30 days, on average, how much time did you spend using short-video platforms per day?

Less than 30 minutes

30–59 minutes

1–2 hours

More than 2 hours but less than 4 hours

4 hours or more

**ACTIVE** Over the past 30 days, have you actively followed or searched for accounts that primarily publish AI content?

Never

Rarely

Sometimes

Often

Always

**EXPOSURE** Over the past 30 days, approximately how frequently did you encounter AI-generated short videos?

Less than once a week

1–2 times a week

3–5 times a week

Almost once a day

Multiple times a day

**CONTENT** What types of content do you primarily watch on short-video platforms? [Multiple choice; select all that apply]

CONTENT1 Entertainment, comedy, or drama.

CONTENT2 Knowledge, science popularization, or education.

CONTENT3 News, society, or current affairs.

CONTENT4 Lifestyle, food, or travel.

CONTENT5 Beauty, fashion, or consumer recommendations.

CONTENT6 Gaming, animation, or movies/TV shows.

CONTENT7 Emotions, psychology, or interpersonal relationships.

CONTENT8 Other, please specify: ____________________

*Each CONTENT option is coded as an independent 0/1 indicator in the dataset.*

**Part 4. Feelings when watching AI-generated short videos in the past 7 days**

*Important instruction: Please refocus your attention on AI-generated short videos. The following items refer to your actual feelings when encountering AI-generated short videos on your selected platform during the past 7 days, not your feelings toward general short videos or overall platform use.*

*Response scale: 1 = Strongly disagree; 2 = Disagree; 3 = Somewhat disagree; 4 = Neither agree nor disagree; 5 = Somewhat agree; 6 = Agree; 7 = Strongly agree.*

**AIF1** I find it difficult to sustain my attention when encountering AI-generated short videos on this platform.

1 2 3 4 5 6 7

**AIF2** I feel cognitively exhausted when browsing this type of content.

1 2 3 4 5 6 7

**AIF3** After encountering this type of content for a while, I find it difficult to continue thinking clearly.

1 2 3 4 5 6 7

**AIF4** Repeatedly encountering AI-generated short videos on this platform makes me feel bored.

1 2 3 4 5 6 7

**AIF5** I sometimes feel irritated when seeing this type of content.

1 2 3 4 5 6 7

**AIF6** Continuously encountering this type of content drains my viewing interest.

1 2 3 4 5 6 7

**SAT1** Overall, I am satisfied with my experience watching AI-generated short videos on this platform over the past 7 days.

1 2 3 4 5 6 7

**SAT2** My experience watching AI-generated short videos over the past 7 days generally met my expectations.

1 2 3 4 5 6 7

**SAT3** Taken together, I hold a positive evaluation of my experience watching AI-generated short videos on this platform over the past 7 days.

1 2 3 4 5 6 7

**CVI1** I am willing to continue watching AI-generated short videos in the next 30 days.

1 2 3 4 5 6 7

**CVI2** When this platform recommends AI-generated short videos to me, I am willing to choose to watch them.

1 2 3 4 5 6 7

**CVI3** I am willing to invest time in watching AI-generated short videos in the next 30 days.

1 2 3 4 5 6 7

**Part 5. General short-video use experience in the past 7 days**

*The following items do not distinguish whether the content is AI-generated. Please respond based on your overall feelings when using your selected short-video platform during the past 7 days.*

*Response scale: 1 = Strongly disagree; 2 = Disagree; 3 = Somewhat disagree; 4 = Neither agree nor disagree; 5 = Somewhat agree; 6 = Agree; 7 = Strongly agree.*

**GSVF1** Over the past 7 days, I often found it difficult to maintain attention for long periods while browsing various short videos on this platform.

1 2 3 4 5 6 7

**GSVF2** After using this platform for a while, I sometimes feel cognitively exhausted.

1 2 3 4 5 6 7

**GSVF3** Prolonged exposure to various types of content on this platform causes me mental fatigue.

1 2 3 4 5 6 7

**GSVF4** Repeatedly browsing content on this platform sometimes gives me a sense of boredom.

1 2 3 4 5 6 7

**GSVF5** I occasionally feel irritable or impatient when using this platform.

1 2 3 4 5 6 7

**GSVF6** Continuously browsing content on this platform diminishes my viewing interest.

1 2 3 4 5 6 7

**Part 6. General attitudes toward artificial intelligence**

*The following items ask about your general views on artificial intelligence, not limited to short videos.*

*Response scale: 1 = Strongly disagree; 2 = Disagree; 3 = Somewhat disagree; 4 = Neither agree nor disagree; 5 = Somewhat agree; 6 = Agree; 7 = Strongly agree.*

**AIATT1** Overall, I am worried that artificial intelligence may bring negative impacts.

1 2 3 4 5 6 7

**AIATT2** I feel uneasy as artificial intelligence becomes increasingly widespread in its usage.

1 2 3 4 5 6 7

**AIATT3** I am concerned that the development of artificial intelligence may exceed effective human control.

1 2 3 4 5 6 7

**AIATT4** Overall, I hold a cautious attitude toward the development of artificial intelligence.

1 2 3 4 5 6 7

**AIATT5** I also acknowledge that artificial intelligence has positive potential in enhancing content-production efficiency.

1 2 3 4 5 6 7

**AC2_Check** To confirm that you have read the instructions for this question, please select “Somewhat agree”.

1 2 3 4 5 6 7

*Researcher coding note (not shown to participants): AIATTneg was calculated as the mean of AIATT1–AIATT4. AIATT5 was excluded from AIATTneg because it measured perceived positive potential rather than negative AI attitude. No reverse coding was applied to AIATT1–AIATT4.*

**Part 7. Basic demographics**

**AGE** What is your age?

18–24 years old

25–34 years old

35–44 years old

45–54 years old

55 years old and above

Prefer not to answer

**GENDER** What is your gender?

Male

Female

Other or self-describe: ____________________

Prefer not to answer

**EDU** What is your highest level of education completed?

High school, vocational school, or below

Associate degree (junior college)

Bachelor’s degree

Master’s degree

Doctoral degree or above

Prefer not to answer

**AREA** What type of area do you currently primarily reside in?

Municipality directly under the Central Government or provincial capital

Other prefecture-level city

County-level city or county

Township or rural area

Prefer not to answer

Thank you for your participation and support. Your responses will help us better understand the user experience of AI-generated content on short-video platforms.
