# Supplementary material for: Perceived AIGC saturation and AI content fatigue on short-video platforms: An appraisal-based structural equation modeling study
Source: PLoS One. 2026 Oct 16;21(10):e0360731. doi: 10.1371/journal.pone.0360731 (PMC13674767; doi:10.1371/journal.pone.0360731)
Supplement: Supplementary file 2 [file pone.0360731.s004.docx]

**Supplementary Tables S1–S10**

Perceived AIGC Saturation and AI Content Fatigue on Short-Video Platforms

**Supplementary Table S1. Item sources and retention decisions**

| Construct | Items | Source/development | Final decision |
| --- | --- | --- | --- |
| PAS | PAS1–PAS4 | Newly developed for perceived AIGC saturation | Retained |
| PLQ | PLQ1–PLQ7 | Newly developed for perceived low-quality prevalence | Retained |
| PLQ | PLQ8 | Candidate item concerning perceived informational value | Removed before formal administration |
| PNH | PNH1–PNH4 | Newly developed for perceived narrative homogenization | Retained |
| PCO | PCO1–PCO4 | Adapted from the information-overload dimension of the Technology Overload Scale [34] | Retained after wording refinement |
| AIF | AIF1–AIF6 | Adapted from cognitive and emotional dimensions of the Social Media Fatigue Scale [13] | Retained after wording refinement |
| SAT | SAT1–SAT3 | Adapted from satisfaction measures based on the expectation-confirmation model [35] | Retained |
| CVI | CVI1–CVI3 | Adapted from continuance-intention measures based on the expectation-confirmation model [35] | Retained |
| GSVF | GSVF1–GSVF6 | Context-adapted general short-video fatigue items | Reported as an adapted covariate measure |
| AIATTneg | AIATT1–AIATT4 | Negative-AI-attitude items; the positive-potential item AIATT5 was not included | Averaged as AIATTneg |

*Note. PLQ8 was removed before formal administration on conceptual grounds; the administered questionnaire retained PLQ1–PLQ7. No data-driven item deletion was conducted after formal administration.*

**Supplementary Table S2. Complete latent-correlation matrix**

| Construct | PAS | PLQ | PNH | PCO | AIF | SAT | CVI |
| --- | --- | --- | --- | --- | --- | --- | --- |
| PAS | 1.000 | 0.504 | 0.425 | 0.386 | 0.455 | -0.270 | -0.271 |
| PLQ | 0.504 | 1.000 | 0.786 | 0.484 | 0.691 | -0.451 | -0.466 |
| PNH | 0.425 | 0.786 | 1.000 | 0.455 | 0.584 | -0.346 | -0.401 |
| PCO | 0.386 | 0.484 | 0.455 | 1.000 | 0.612 | -0.327 | -0.351 |
| AIF | 0.455 | 0.691 | 0.584 | 0.612 | 1.000 | -0.540 | -0.550 |
| SAT | -0.270 | -0.451 | -0.346 | -0.327 | -0.540 | 1.000 | 0.879 |
| CVI | -0.271 | -0.466 | -0.401 | -0.351 | -0.550 | 0.879 | 1.000 |

*Note. Values are standardized latent correlations from the primary seven-factor CFA. In the supplementary GSVF model, AIF–GSVF = .742 and PCO–GSVF = .431.*

**Supplementary Table S3. Complete HTMT matrix**

| Construct | PAS | PLQ | PNH | PCO | AIF | SAT | CVI | GSVF |
| --- | --- | --- | --- | --- | --- | --- | --- | --- |
| PAS | — | 0.501 | 0.423 | 0.381 | 0.454 | 0.268 | 0.268 | 0.347 |
| PLQ | 0.501 | — | 0.785 | 0.480 | 0.690 | 0.453 | 0.465 | 0.466 |
| PNH | 0.423 | 0.785 | — | 0.456 | 0.583 | 0.346 | 0.400 | 0.413 |
| PCO | 0.381 | 0.480 | 0.456 | — | 0.608 | 0.325 | 0.348 | 0.429 |
| AIF | 0.454 | 0.690 | 0.583 | 0.608 | — | 0.540 | 0.547 | 0.741 |
| SAT | 0.268 | 0.453 | 0.346 | 0.325 | 0.540 | — | 0.878 | 0.451 |
| CVI | 0.268 | 0.465 | 0.400 | 0.348 | 0.547 | 0.878 | — | 0.422 |
| GSVF | 0.347 | 0.466 | 0.413 | 0.429 | 0.741 | 0.451 | 0.422 | — |

*Note. All HTMT values were below .90. Focal values were PCO–AIF=.608, AIF–GSVF=.741, PCO–GSVF=.429, and SAT–CVI=.878.*

**Supplementary Table S4. One-factor versus two-factor CFA comparisons**

| Construct pair | Model | χ² (df) | CFI | RMSEA | SRMR | Δχ² (df) | p | ΔCFI |
| --- | --- | --- | --- | --- | --- | --- | --- | --- |
| PCO–AIF | One-factor | 980.848 (419) | .937 | .051 | .045 | — | — | — |
| PCO–AIF | Two-factor | 462.662 (413) | .994 | .015 | .028 | — | — | — |
| PCO–AIF | Difference | — | — | — | — | 518.186 (6) | <.001 | .057 |
| AIF–GSVF | One-factor | 1185.692 (608) | .947 | .043 | .045 | — | — | — |
| AIF–GSVF | Two-factor | 682.119 (601) | .993 | .016 | .028 | — | — | — |
| AIF–GSVF | Difference | — | — | — | — | 503.573 (7) | <.001 | .046 |

*Note. The two-factor models fit substantially better than their corresponding one-factor models.*

**Supplementary Table S5. GSVF item wording and measurement results**

| Item | Wording | Standardized loading |
| --- | --- | --- |
| GSVF1 | Over the past 7 days, I often found it difficult to maintain attention for long periods while browsing various short videos on this platform. | .757 |
| GSVF2 | After using this platform for a while, I sometimes feel cognitively exhausted. | .783 |
| GSVF3 | Prolonged exposure to various types of content on this platform causes me mental fatigue. | .774 |
| GSVF4 | Repeatedly browsing content on this platform sometimes gives me a sense of boredom. | .753 |
| GSVF5 | I occasionally feel irritable or impatient when using this platform. | .758 |
| GSVF6 | Continuously browsing content on this platform diminishes my viewing interest. | .769 |

*Note. GSVF reliability: Cronbach's α=.895; McDonald's ω=.895; composite reliability=.895; AVE=.586.*

**Supplementary Table S6. Complete covariate path estimates in the covariate-adjusted structural model**

| Covariate | Outcome | Standardized β | ML p | Bootstrap p | Bias-corrected 95% CI |
| --- | --- | --- | --- | --- | --- |
| GSVF | AIF | .527 | <.001 | .001 | [.445,.604] |
| GSVF | SAT | −.002 | .974 | .871 | [−.232,.216] |
| GSVF | CVI | .057 | .406 | .684 | [−.170,.269] |
| AIATTneg | AIF | .032 | .293 | .322 | [−.031,.095] |
| AIATTneg | SAT | .060 | .171 | .253 | [−.040,.150] |
| AIATTneg | CVI | .035 | .422 | .451 | [−.056,.131] |
| CONF | AIF | .026 | .388 | .447 | [−.043,.096] |
| CONF | SAT | .016 | .717 | .770 | [−.080,.106] |
| CONF | CVI | .084 | .055 | .064 | [−.005,.178] |
| EXPOSURE | AIF | −.009 | .762 | .762 | [−.072,.052] |
| EXPOSURE | SAT | .111 | .011 | .016 | [.020,.200] |
| EXPOSURE | CVI | .118 | .007 | .013 | [.023,.207] |
| USE | AIF | .041 | .173 | .201 | [−.021,.101] |
| USE | SAT | .008 | .854 | .879 | [−.076,.090] |
| USE | CVI | .077 | .076 | .090 | [−.010,.164] |
| ACTIVE | AIF | .042 | .165 | .186 | [−.020,.103] |
| ACTIVE | SAT | .032 | .465 | .534 | [−.061,.119] |
| ACTIVE | CVI | .010 | .828 | .830 | [−.079,.106] |
| age_25_34 | AIF | −.068 | .062 | .090 | [−.144,.011] |
| age_35_44 | AIF | −.089 | .010 | .022 | [−.160,−.014] |
| age_45_54 | AIF | −.006 | .852 | .887 | [−.080,.071] |
| age_55plus | AIF | −.024 | .449 | .433 | [−.088,.039] |
| age_pna | AIF | .021 | .497 | .667 | [−.064,.083] |
| edu_assoc | AIF | −.005 | .917 | .914 | [−.098,.085] |
| edu_bachelor | AIF | .030 | .520 | .565 | [−.068,.120] |
| edu_master | AIF | .033 | .385 | .397 | [−.043,.103] |
| edu_doctoral | AIF | −.086 | .006 | .001 | [−.154,−.026] |
| edu_pna | AIF | .010 | .741 | .639 | [−.032,.051] |
| area_county | AIF | −.056 | .243 | .247 | [−.154,.041] |
| area_prefecture | AIF | −.007 | .899 | .919 | [−.125,.112] |
| area_municipality | AIF | .003 | .962 | .954 | [−.114,.125] |
| area_pna | AIF | −.009 | .789 | .787 | [−.067,.052] |
| gender_female | AIF | .037 | .227 | .260 | [−.027,.102] |
| gender_other | AIF | −.061 | .044 | .013 | [−.112,−.012] |
| gender_pna | AIF | .022 | .464 | .477 | [−.039,.081] |
| age_25_34 | SAT | −.060 | .263 | .331 | [−.175,.062] |
| age_25_34 | CVI | .055 | .304 | .331 | [−.058,.172] |
| age_35_44 | SAT | .024 | .638 | .640 | [−.087,.137] |
| age_35_44 | CVI | .000 | .999 | .939 | [−.106,.111] |
| age_45_54 | SAT | .005 | .919 | .913 | [−.098,.106] |
| age_45_54 | CVI | .058 | .239 | .304 | [−.050,.165] |
| age_55plus | SAT | .007 | .883 | .859 | [−.085,.101] |
| age_55plus | CVI | .056 | .234 | .232 | [−.034,.148] |
| age_pna | SAT | .003 | .938 | .915 | [−.095,.116] |
| age_pna | CVI | .025 | .575 | .683 | [−.097,.170] |
| edu_assoc | SAT | −.032 | .611 | .684 | [−.173,.110] |
| edu_assoc | CVI | −.021 | .737 | .746 | [−.152,.111] |
| edu_bachelor | SAT | −.030 | .658 | .749 | [−.176,.133] |
| edu_bachelor | CVI | .001 | .993 | .995 | [−.138,.145] |
| edu_master | SAT | −.021 | .697 | .746 | [−.147,.111] |
| edu_master | CVI | .023 | .671 | .718 | [−.091,.131] |
| edu_doctoral | SAT | −.056 | .218 | .219 | [−.135,.030] |
| edu_doctoral | CVI | −.010 | .830 | .821 | [−.081,.060] |
| edu_pna | SAT | .003 | .953 | .920 | [−.053,.064] |
| edu_pna | CVI | .019 | .681 | .668 | [−.058,.081] |
| area_county | SAT | −.038 | .584 | .604 | [−.185,.117] |
| area_county | CVI | −.034 | .631 | .693 | [−.178,.123] |
| area_prefecture | SAT | −.131 | .118 | .168 | [−.311,.057] |
| area_prefecture | CVI | −.068 | .421 | .497 | [−.245,.123] |
| area_municipality | SAT | −.109 | .200 | .261 | [−.290,.084] |
| area_municipality | CVI | −.118 | .167 | .218 | [−.301,.067] |
| area_pna | SAT | −.034 | .471 | .340 | [−.110,.037] |
| area_pna | CVI | −.064 | .188 | .234 | [−.164,.043] |
| gender_female | SAT | .022 | .619 | .670 | [−.074,.112] |
| gender_female | CVI | .003 | .944 | .960 | [−.093,.100] |
| gender_other | SAT | .055 | .204 | .137 | [−.017,.129] |
| gender_other | CVI | .015 | .735 | .620 | [−.045,.085] |
| gender_pna | SAT | .000 | .995 | .978 | [−.091,.099] |
| gender_pna | CVI | .096 | .031 | .018 | [.016,.183] |

*Note. Bootstrap p values and intervals are bias-corrected results from 5,000 usable bootstrap samples. Demographic reference categories were 18–24 years, high school/vocational school or below, township/rural area, and male.*

**Supplementary Table S7. Direct-path sensitivity analysis**

| Direct path | Unstandardized estimate | SE | Standardized β | p | 95% CI |
| --- | --- | --- | --- | --- | --- |
| PAS → AIF | −.0106 | .1133 | −.0054 | .925 | [−.2327,.2115] |
| PAS → SAT | −.0690 | .0744 | −.0568 | .354 | [−.2148,.0768] |
| PAS → CVI | −.0523 | .0761 | −.0425 | .492 | [−.2015,.0968] |

*Note. The sensitivity and comparison models were estimated using the same R/lavaan specification. The primary adjusted structural model was estimated in AMOS; therefore, its fit indices were not used in the Δχ² comparison.*

**Supplementary Table S8. Unadjusted structural-model estimates**

| Path | Standardized β | ML p | Bootstrap p | Conclusion |
| --- | --- | --- | --- | --- |
| PAS → PLQ | .581 | <.001 | .001 | Supported |
| PAS → PNH | .515 | <.001 | <.001 | Supported |
| PAS → PCO | .447 | <.001 | .001 | Supported |
| PLQ → AIF | .500 | <.001 | <.001 | Supported |
| PNH → AIF | .105 | .010 | .051 | Not supported |
| PCO → AIF | .374 | <.001 | <.001 | Supported |
| AIF → SAT | −.566 | <.001 | .001 | Supported |
| AIF → CVI | −.578 | <.001 | <.001 | Supported |

*Note. Unadjusted model fit: 70 estimated parameters; χ²(426)=1007.883; CFI=.935; TLI=.929; IFI=.935; RMSEA=.052; 90% CI [.048,.056]. This model is reported for comparison; the covariate-adjusted model is the primary structural model.*

| Endogenous construct | R² |
| --- | --- |
| PLQ | .338 |
| PNH | .265 |
| PCO | .200 |
| AIF | .547 |
| SAT | .320 |
| CVI | .334 |

**Supplementary Table S9. Sample characteristics of valid respondents (N = 513)**

| Variable | Category | n | % |
| --- | --- | --- | --- |
| Residence in mainland China | Yes | 513 | 100.00% |
| Use days per week | 3–4 days | 89 | 17.35% |
| Use days per week | 5–6 days | 120 | 23.39% |
| Use days per week | Every day | 304 | 59.26% |
| Most-used platform | Douyin | 293 | 57.12% |
| Most-used platform | Kuaishou | 80 | 15.59% |
| Most-used platform | WeChat Channels | 42 | 8.19% |
| Most-used platform | Bilibili | 49 | 9.55% |
| Most-used platform | Xiaohongshu | 49 | 9.55% |
| AI short-video exposure | Yes | 513 | 100.00% |
| AI-identification confidence | 1 | 7 | 1.36% |
| AI-identification confidence | 2 | 15 | 2.92% |
| AI-identification confidence | 3 | 60 | 11.70% |
| AI-identification confidence | 4 | 173 | 33.72% |
| AI-identification confidence | 5 | 169 | 32.94% |
| AI-identification confidence | 6 | 66 | 12.87% |
| AI-identification confidence | 7 | 23 | 4.48% |
| Daily use intensity | <30 min | 22 | 4.29% |
| Daily use intensity | 30–59 min | 86 | 16.76% |
| Daily use intensity | 1–2 h | 203 | 39.57% |
| Daily use intensity | 2–4 h | 150 | 29.24% |
| Daily use intensity | ≥4 h | 52 | 10.14% |
| Active AI-account following/searching | Never | 151 | 29.43% |
| Active AI-account following/searching | Rarely | 190 | 37.04% |
| Active AI-account following/searching | Sometimes | 93 | 18.13% |
| Active AI-account following/searching | Often | 47 | 9.16% |
| Active AI-account following/searching | Always | 32 | 6.24% |
| AI-content exposure frequency | Almost once a day | 22 | 4.29% |
| AI-content exposure frequency | Less than once a week | 80 | 15.59% |
| AI-content exposure frequency | 1–2 times per week | 87 | 16.96% |
| AI-content exposure frequency | 3–5 times per week | 149 | 29.04% |
| AI-content exposure frequency | Multiple times a day | 175 | 34.11% |
| LOWQ judgment frequency | Never | 45 | 8.77% |
| LOWQ judgment frequency | Rarely | 121 | 23.59% |
| LOWQ judgment frequency | Sometimes | 221 | 43.08% |
| LOWQ judgment frequency | Often | 105 | 20.47% |
| LOWQ judgment frequency | Always | 21 | 4.09% |
| Age | 18–24 | 138 | 26.90% |
| Age | 25–34 | 178 | 34.70% |
| Age | 35–44 | 89 | 17.35% |
| Age | 45–54 | 62 | 12.09% |
| Age | 55+ | 36 | 7.02% |
| Age | Prefer not to say | 10 | 1.95% |
| Gender | Male | 256 | 49.90% |
| Gender | Female | 231 | 45.03% |
| Gender | Other | 13 | 2.53% |
| Gender | Prefer not to say | 13 | 2.53% |
| Education | High school/vocational or below | 68 | 13.26% |
| Education | Associate degree | 119 | 23.20% |
| Education | Bachelor degree | 255 | 49.71% |
| Education | Master degree | 55 | 10.72% |
| Education | Doctoral degree or above | 9 | 1.75% |
| Education | Prefer not to say | 7 | 1.36% |
| Residential area | Township/rural | 38 | 7.41% |
| Residential area | County-level city | 79 | 15.40% |
| Residential area | Prefecture-level city | 186 | 36.26% |
| Residential area | Municipality/provincial capital | 200 | 38.99% |
| Residential area | Prefer not to say | 10 | 1.95% |

*Note. Participants were recruited through Wenjuanxing, WeChat, Xiaohongshu, QQ, and related online networks. No monetary or material compensation was provided. Percentages are based on N=513 and may not sum to exactly 100% because of rounding.*

**Supplementary Table S10. Participant flow and exclusion summary**

| Screening stage | n | % of initial sample |
| --- | --- | --- |
| Initial responses | 536 | 100.00% |
| AC1 passed | 529 | 98.69% |
| AC1 failed | 7 | 1.31% |
| AC2 passed | 520 | 97.01% |
| AC2 failed | 16 | 2.99% |
| Failed both AC1 and AC2 | 0 | 0.00% |
| Failed AC1 only | 7 | 1.31% |
| Failed AC2 only | 16 | 2.99% |
| Additional exclusions: core-item missingness | 0 | 0.00% |
| Additional exclusions: completion time | 0 | 0.00% |
| Additional exclusions: long-string responding | 0 | 0.00% |
| Additional exclusions: contradictory responses | 0 | 0.00% |
| Additional exclusions: duplicate records | 0 | 0.00% |
| Final valid sample | 513 | 95.71% |

*Note. AC1 required “2 = Disagree,” and AC2 required “5 = Somewhat agree.” The two attention-check failures did not overlap. Eligibility and attention-check rules were defined before inspecting survey responses and before confirmatory analyses. Valid-response duration: median 590 s, Q1=516 s, Q3=673 s, range 60–904 s (IQR=157 s). The AC1/AC2 pass/fail rows are descriptive and are not additive.*
