# Supplementary material for: Perceived AIGC saturation and AI content fatigue on short-video platforms: An appraisal-based structural equation modeling study
Source: PLoS One. 2026 Oct 16;21(10):e0360731. doi: 10.1371/journal.pone.0360731 (PMC13674767; doi:10.1371/journal.pone.0360731)
Supplement: Supplementary file 3 [file pone.0360731.s006.docx]

**Response to the Academic Editor and Reviewers**

Manuscript ID: PONE-D-26-35682

Perceived AIGC saturation and AI content fatigue on short-video platforms

Dear Dr. Donghwa Chung,

Thank you for your careful editorial assessment of our manuscript and for the opportunity to submit a revised version. We also sincerely thank the two anonymous reviewers for their careful reading, constructive criticism, and substantial effort. We have considered all of the comments carefully and agree that they helped improve the transparency, conceptual consistency, statistical reporting, and reproducibility of the manuscript. We have addressed the comments point by point in the responses below, and all corresponding changes are marked in the revised manuscript.

## Editorial comments

Response: We have clarified the source and development status of every measurement instrument, expanded the description of the item-generation and expert-review process, separated the independent CFA from the adjusted structural model, corrected the reported fit statistics, updated the ethics statement, and aligned the conceptual definitions across the Introduction, hypothesis development, Measures, Results, Discussion, and Conclusion.

We have also added the following acknowledgement to the revised manuscript: We thank Academic Editor Dr. Donghwa Chung and the two anonymous reviewers for their careful evaluation and constructive suggestions, which helped improve this manuscript.

The detailed responses to Reviewer 1 and Reviewer 2 follow. We hope that the revised manuscript and the accompanying explanations address the concerns raised during review.

## Reviewer 1

## Comment 1

**Response:** Thank you for identifying this important omission. We agree that the original manuscript did not sufficiently describe the questionnaire-development process. We have therefore expanded the Methods section to clarify the sources and generation of each construct’s items, the language and adaptation procedures, the expert-review process, the evaluation dimensions, and the item-retention decisions. The questionnaire was administered in Simplified Chinese. PAS, PLQ, and PNH were newly developed from their operational definitions and the AIGC short-video context. PCO items were adapted from the information-overload dimension of the Technology Overload Scale, AIF items were adapted from the cognitive and emotional dimensions of the Social Media Fatigue Scale, and SAT and CVI items were adapted from satisfaction and continuance-intention measures based on the expectation-confirmation model. Before formal data collection, five experts with backgrounds in media psychology, communication and human–computer interaction, psychometrics and statistics, survey methodology, and short-video industry/content governance independently reviewed the items in one review round. They evaluated relevance, clarity, comprehensibility, and contextual appropriateness. The research team then consolidated the feedback and implemented the wording revisions. No separate participant pilot study or formal cognitive interview was conducted. PLQ8 was removed before formal administration because it assessed perceived informational value rather than the audiovisual, logical, and production-quality aspects defined by the PLQ construct. The final administered questionnaire therefore contained PLQ1–PLQ7. The item sources, wording changes, and retention decisions are now documented in the revised Methods section and Supplementary Table S1. The final questionnaire and coding information are also provided in the supplementary materials.

## Comment 2

**Response:** Thank you for raising this important concern. We agree that the phrase “psychometrically developed questionnaire” overstated the level of evidence provided by the present study. The seven-factor measurement structure for the core constructs—PAS, PLQ, PNH, PCO, AIF, SAT, and CVI—was specified before formal data collection on the basis of the construct definitions, item-generation rationale, and expert review. However, the factor structure was not developed through an exploratory factor analysis in the present dataset, and no separate pilot, development sample, or independent CFA sample was available. All 513 valid responses were used for the CFA and subsequent SEM analyses. We have therefore replaced “psychometrically developed questionnaire” with “theory-informed, context-specific questionnaire” and now describe the CFA results as preliminary measurement evidence rather than independent scale validation. We have also added a limitation stating that the measurement structure requires replication using an independent sample and, ideally, a separate EFA–CFA sequence. PLQ8 was removed before formal administration on conceptual grounds following expert review; no item was deleted after inspecting the CFA results from the 513 respondents.

## Comment 3

**Response:** Thank you for this helpful suggestion. We agree that the original manuscript provided only a summary statement about HTMT values and did not show the complete discriminant-validity evidence. We have now added the complete latent-correlation matrix and HTMT matrix to the Supplementary Materials. In the seven-factor CFA, the largest latent correlation was between SAT and CVI (r = .879). The latent correlation between PCO and AIF was r = .612. In the supplementary model including general short-video fatigue (GSVF), the latent correlations were r = .742 for AIF–GSVF and r = .431 for PCO–GSVF. The corresponding HTMT values were .608, .741, and .429, respectively. All HTMT values were below .90. We also conducted nested one-factor versus two-factor CFA comparisons. For PCO and AIF, the two-factor model fit substantially better than the one-factor model, Δχ²(6) = 518.186, p < .001, ΔCFI = .057. For AIF and GSVF, the two-factor model also fit substantially better, Δχ²(7) = 503.573, p < .001, ΔCFI = .046. These results provide additional evidence that PCO, AIF, and GSVF represent empirically distinguishable constructs, although their conceptual relatedness is acknowledged.

## Comment 4

**Response:** Thank you for pointing out this omission. We agree that the original manuscript mentioned general short-video fatigue (GSVF) as a control construct but did not report its measurement properties or its role in the structural model. We have now added the complete GSVF item wording and coding information to the Supplementary Materials. GSVF was measured using six context-adapted items covering attention difficulty, cognitive tiredness, mental fatigue, boredom, irritation, and reduced viewing interest during general short-video use. The six GSVF items showed standardized loadings ranging from .753 to .783. Internal consistency was satisfactory (Cronbach’s α = .895; McDonald’s ω = .895), with composite reliability of .895 and AVE of .586. The latent correlation between GSVF and AIF was r = .742, and their HTMT value was .741. The two-factor model distinguishing AIF from GSVF fit substantially better than the one-factor model. After controlling for GSVF and the other covariates, AIF remained negatively associated with satisfaction (β = −.575, p < .001) and continued viewing intention (β = −.629, p < .001). These findings indicate that AI content fatigue retains explanatory value beyond general short-video fatigue.

## Comment 5

**Response:** Thank you for identifying this important reporting problem. We re-checked the archived AMOS outputs and confirmed that the original manuscript inadvertently reported the same fit statistics for the CFA and structural model. We have corrected this error and now report the two models separately. The measurement model was a freely correlated seven-factor CFA including PAS, PLQ, PNH, PCO, AIF, SAT, and CVI. It contained 83 estimated parameters and 413 degrees of freedom. The adjusted structural model included the measurement component, structural paths, GSVF, AIATTneg, exposure and usage controls, and demographic covariates. It contained 449 estimated parameters and 1,321 degrees of freedom. The revised manuscript now reports the model specifications, parameter counts, degrees of freedom, and fit indices separately. The corresponding AMOS output files are provided in the supplementary analysis materials.

## Comment 6

**Response:** Thank you for requesting these methodological details. We have expanded the Statistical analysis section to clarify the software, estimator, treatment of the Likert-scale items, missing-data handling, distributional checks, factor scaling, bootstrap procedure, residual specification, and model-modification procedure. The CFA and SEM analyses were conducted using IBM SPSS Amos26 with maximum likelihood estimation. The seven-point Likert items were treated as approximately continuous variables because they had seven response categories and showed limited skewness and kurtosis. Item-level skewness ranged from −.177 to .188, and excess kurtosis ranged from −.425 to .323. No missing core-item values were present in the final analytic sample, and no imputation was performed. The first loading of each latent factor was fixed to 1.0 for identification. No correlated indicator residuals were specified, and no post hoc structural paths or residual covariances were added on the basis of modification indices. Robust standard errors and scaled fit indices were not used in the AMOS maximum-likelihood analyses. Indirect effects were evaluated using 5,000 usable bootstrap samples and bias-corrected 95% confidence intervals. The revised manuscript now reports SRMR separately from AMOS’s RMR. The AMOS output for the adjusted structural model showed a standardized RMR of .0696. The SRMR for the matched supplementary lavaan CFA was .028.

## Comment 7

**Response:** Thank you for identifying this inconsistency. We agree that the original manuscript did not clearly distinguish the unadjusted and covariate-adjusted structural models, and that the presentation of the R² values could suggest that the covariates had not been included.

We have now estimated and reported two structural specifications. The unadjusted model included only the hypothesized paths among PAS, PLQ, PNH, PCO, AIF, SAT, and CVI. The covariate-adjusted model additionally included GSVF, AIATTneg, CONF, EXPOSURE, USE, ACTIVE, and dummy-coded age, gender, education, and residential-area variables. Each covariate was specified as an observed exogenous predictor of AIF, SAT, and CVI. The covariate-adjusted model is treated as the primary structural model.

The unadjusted model had χ²(426) = 1007.883, CFI = .935, TLI = .929, and RMSEA = .052. The adjusted model had χ²(1321) = 1991.541, CFI = .946, TLI = .929, RMSEA = .031, and standardized RMR = .0696.

In the adjusted model, the R² values were .364 for PLQ, .288 for PNH, .216 for PCO, .747 for AIF, .351 for SAT, and .374 for CVI. These values are the squared multiple correlations obtained from the covariate-adjusted model.

The complete coefficients for the hypothesized paths and all covariate paths are now reported in Table 2 and Supplementary Table S6. Figure 2 has been revised to display the focal structural paths and the GSVF control paths. The remaining covariate paths are reported in full in Supplementary Table S6 and were omitted from the figure for visual clarity. The previously reported coefficients and R² values are now explicitly labelled as results from the unadjusted model.

## Comment 8

**Response:** Thank you for identifying this inconsistency. We agree that the previous Table 4 mixed path-specific indirect effects with shorter component-specific indirect paths. We have now reorganized the indirect-effect analysis according to the prespecified hypotheses.

H4a–H4c represent PAS → appraisal mechanism → AIF. H5a–H5c represent PAS → appraisal mechanism → AIF → SAT. H6a–H6c represent PAS → appraisal mechanism → AIF → CVI. For each pathway, we now report the unstandardized estimate, standardized estimate, bootstrap SE, bias-corrected 95% confidence interval, and bootstrap p value.

The revised results show that the indirect pathways through PLQ and PCO were supported, whereas the corresponding pathways through PNH were not supported. We also clarify that significance of an overall indirect effect does not establish significance of every individual component pathway.

## Comment 9

**Response:** Thank you for raising this important point. We agree that the original manuscript did not sufficiently explain why the primary model omitted direct paths from PAS to AIF, SAT, and CVI.

The primary model was specified as an indirect-only model because the theoretical framework focuses on appraisal-based mechanisms. Specifically, perceived AIGC saturation was expected to be associated with downstream outcomes through perceived low-quality prevalence, perceived narrative homogenization, perceived content overload, and AI content fatigue. The omission of direct paths was therefore theory-driven and was not intended to imply that direct associations were impossible.

To assess the robustness of this specification, we estimated a sensitivity model using R/lavaan by adding direct paths from PAS to AIF, SAT, and CVI while retaining the hypothesized paths and covariates. Both the sensitivity model and its comparison model used the same 513 respondents, variables, estimator, and model parameterization. The additional paths were not significant, and the matched R/lavaan model comparison showed no material improvement in fit, Δχ²(3) = .937, p = .816. This comparison was based solely on the two matched R/lavaan models and was not calculated from the AMOS fit statistics for the primary adjusted structural model. The complete sensitivity results are reported in Supplementary Table S7.

We have clarified that the sensitivity results are consistent with the indirect-only specification, but do not provide definitive proof of complete mediation.

## Comment 10

**Response:** Thank you for identifying this inconsistency. We agree that the previous manuscript mixed maximum-likelihood p values, bootstrap p values, and rounded confidence intervals when interpreting the narrative-homogenization pathway.

We have now specified the bias-corrected 95% bootstrap confidence interval as the primary inferential criterion for indirect effects. A pathway is considered supported only when its confidence interval excludes zero. ML p values are reported as supplementary information and are not used to override the bootstrap-based conclusion.

In the covariate-adjusted model, the PNH → AIF path was β = .036, ML p = .298, bootstrap p = .467, with a bias-corrected 95% CI of [−.052, .116]. Therefore, this path is classified as not supported.

We also replaced rounded confidence limits and report the relevant estimates to at least three decimal places. Any confidence interval that includes zero is now described as not supported, regardless of whether a separate p value is below .05.

## Comment 11

**Response:** Thank you for identifying this inconsistency. We have revised the Methods and Results sections to distinguish prespecified exclusion rules from supplementary data-quality checks.

Of the 536 initial responses, seven failed AC1 only and 16 failed AC2 only. No respondent failed both attention checks. Thus, 23 responses were excluded for failing at least one attention check, and 513 valid responses remained. No additional cases were excluded because of missing core items, completion time, long-string responding, contradictory responses, or duplicate records.

We have added a participant-flow table and reported the completion-time distribution for the final valid sample. The detailed variable coding and screening rules are cross-referenced in Supplementary File S3.

## Comment 12

**Response:** Thank you for this important theoretical clarification. We have revised the manuscript to explain how the three appraisal mechanisms map onto Cognitive Appraisal Theory and the S–O–R framework.

PLQ and PNH are conceptualized as content-focused subjective appraisals of perceived production quality and narrative similarity. PCO is conceptualized as a resource-oriented appraisal of the cognitive demands required to identify, filter, and evaluate AI-generated videos. It does not measure fatigue itself. In the S–O–R model, perceived AIGC saturation functions as the stimulus, PLQ, PNH, and PCO represent organism-level appraisal processes, AI content fatigue represents a subsequent internal state, and satisfaction and continued viewing intention represent downstream responses.

We also clarify that these three mechanisms were selected to represent production-quality, narrative-similarity, and cognitive-processing dimensions of AI short-video experience. Perceived authenticity, deception, trust, novelty loss, and algorithmic intrusiveness are plausible alternative mechanisms, but they were not measured in this study. We now acknowledge this limitation and identify these constructs as directions for future research.

## Comment 13

## **Response:** Thank you for this comment. We have revised the ethics statement to identify the institution and reviewing committee. The protocol was reviewed by the Academic Committee of Jinling Institute of Technology, which confirmed that the project met the institutional criteria for exemption from full ethical review. The exemption statement was issued on May 13, 2026. No separate exemption or approval number was provided by the committee. We have also clarified that informed consent was recorded electronically through a required consent item before participants could access the questionnaire.

## Comment 14

## **Response: Thank you for identifying this inconsistency. We have aligned the Data Availability Statement with the Zenodo record and identified the de-identified dataset, questionnaire, codebook, and supplementary tables deposited there. The AMOS model files, AMOS output files, executable R scripts, and README are included in the companion Zenodo record. in the same record before resubmission and will be included in the final repository description once the updated record is published. These materials are intended to support reproduction of the descriptive statistics, CFA, reliability and validity analyses, adjusted SEM, bootstrap indirect effects, and sensitivity analyses.**

## **Comment 15**

## **Response: Thank you for raising this important issue. We have clarified that the study used an umbrella definition of AIGC. Fully generated videos were defined as videos in which major visual, audio, animation, character, or script elements were generated by generative AI. Substantially AI-assisted videos were defined as videos in which generative AI materially contributed to one or more major audiovisual or script elements.**

## **Participants were not required to classify each video according to the degree of AI involvement. Both fully generated and substantially AI-assisted videos were included when participants could reasonably identify them as AI-related. The questionnaire did not provide standardized video examples; instead, it provided written identification cues, including platform labels, creator disclosures, account information, and visible generative features.**

## **Accordingly, perceived AIGC saturation should be interpreted as perceived saturation of identifiable AI-generated or substantially AI-assisted short videos, rather than as an estimate of the objective prevalence of fully AI-generated content. Potential misclassification may have introduced measurement error. If misclassification was nondifferential, the observed associations may have been attenuated; if it was related to AI attitudes or identification confidence, bias in either direction is possible.**

## **Comment 16**

## **Response: Thank you for this important comment. We agree that several sentences in the Discussion used stronger causal language than warranted by the cross-sectional design. We have replaced terms such as “emerges,” “triggers,” “drives,” and “produces” with association-based wording. We also clarified that the observed direction is theoretically specified but cannot establish temporal ordering or causality. In addition, we acknowledged that fatigue or dissatisfaction may have influenced participants’ negative evaluations of AI-generated content and their reports of perceived AIGC saturation.**

## **Comment 17**

## **Response: Thank you for this suggestion. We have added a complete sample-characteristics table reporting age, gender, education, residential area, most frequently used platform, AI-content exposure frequency, daily short-video use intensity, AI-identification confidence, and active following or searching for AI-content accounts. We have also clarified the recruitment channels, compensation arrangements, and the distinction between eligibility screening before the main questionnaire and post-completion data-quality exclusions.**

## **Of the 536 completed responses, 23 were excluded after completion because they failed at least one attention check, leaving 513 valid responses. The eligibility requirement concerning reasonably identifiable AI-generated content may have preferentially selected participants with greater AI awareness or exposure. We have added this possibility as a potential source of selection bias in the Limitations section.**

## **Comment 18**

## **Response: Thank you for this comment. No formal a priori power calculation or preregistered sample-size target was conducted before data collection. The final sample size was determined by the feasible recruitment of eligible participants. We have removed any wording that could imply that the sample was prospectively powered. Instead, we now describe the complexity of the measurement and structural models and provide a confidence-interval-based precision assessment. These results are presented as preliminary evidence rather than as confirmation of an a priori power calculation.**

## **Comment 19**

## **Response: Thank you for this important comment. We agree that Harman’s single-factor test cannot establish that common-method variance had no substantive influence on the findings. We have therefore revised the manuscript to describe Harman’s test only as a limited supplementary diagnostic.**

## **We now explicitly acknowledge that all core constructs were measured from the same 513 respondents at a single time point, using similarly structured seven-point self-report scales. Several constructs also involved related evaluative or affective wording, which may increase the risk of shared method variance. Although the first unrotated factor accounted for 37.67% of the variance, this result is not interpreted as evidence that common-method bias was absent. The manuscript now states that common-method variance cannot be ruled out and that the findings should be interpreted with this limitation in mind.**

## **Comment 20**

## **Response: Thank you for this important observation. We agree that the three appraisal constructs primarily capture negative evaluations of AI-generated content, which may share evaluative valence with AI content fatigue and dissatisfaction. We have therefore weakened statements suggesting that users do not reject AIGC itself or that negative appraisals are the only relevant mechanisms. We also clarified that positive and neutral evaluations of AIGC were not measured, and that the present model cannot determine whether AI origin itself affects user responses independently of evaluative valence.**

## **Comment 21**

## **Response: Thank you for this clarification. We agree that a larger standardized coefficient does not by itself demonstrate a statistically significant difference between coefficients. Because no formal Wald or bootstrap coefficient-comparison test was conducted, we have removed the wording “strongest predictor” and “strongest mechanism.” We now describe perceived low-quality prevalence as having the largest estimated standardized coefficient among the three appraisal paths, without claiming that it was statistically stronger than the other predictors.**

## **Comment 22**

## **Response: Thank you for this important clarification. We agree that the original practical recommendations extended beyond the interventions tested in this study. We have revised the Practical implications section to present granular AIGC labels, recommendation controls, automated duplicate detection, quality-sensitive moderation, and prioritization of human-created content as tentative design proposals for future research. We now explicitly state that their effectiveness was not tested in the present study.**

## **Comment 23**

## **Response: Thank you for recommending these recent studies. We have considered and cited both articles in the revised Discussion.**

## **The recent AI-fatigue study provides a useful conceptual and measurement benchmark by treating AI fatigue as a multidimensional construct involving cognitive, emotional, behavioral, and physical components [46]. We now cite this work to situate the present AIF construct, while clarifying that our study focuses specifically on cognitive and affective fatigue associated with AI-generated short-video content.**

## **We also cited the recent study on critical thinking in generative AI use, which emphasizes verification, epistemic motivation, and reflective judgment as dimensions of critical engagement with AI outputs [47]. This work supports our discussion of the cognitive effort involved in identifying, filtering, and evaluating AIGC.**

## **Because these studies were published after our data collection and were not part of the original questionnaire, we did not retroactively adopt their scales or alter the current analyses. Instead, we present them as recent conceptual and measurement references and as directions for future research.**

## **Comment 24**

## **Response: Thank you for this important comment. We agree that the original wording overinterpreted the weak result for perceived narrative homogenization. Because the bootstrap confidence interval included zero, we no longer describe this finding as “theoretically meaningful” or “marginally robust.” We now report the result as statistically unsupported and acknowledge that the estimate may be sensitive to measurement overlap among appraisal constructs, shared-variance suppression or redistribution, and sampling uncertainty.**

## ****Reviewer 2,****

## **Comment 1**

## **Response: Thank you for this helpful suggestion. We agree that the previous version repeated the definitions of the three appraisal constructs across several sections. We have reorganized the manuscript to clarify the progression from concepts to hypotheses and measurement. The Introduction now provides only concise definitions of PLQ, PNH, and PCO. The hypothesis-development section focuses on their theoretical roles within Cognitive Appraisal Theory and the S–O–R framework. The Measures section now reports only item numbers, referent period, item sources, response format, and coding information. Repetitive construct definitions have been removed from the Discussion and Conclusion.**

## **Comment 2**

## **Response: Thank you for this important comment. We agree that the development of the AIGC-specific measures required a more transparent description. We have now clarified the item-generation process, theoretical basis, expert review, item revision and deletion decisions, absence of participant pilot testing, and final item-retention procedure.**

## **PAS, PLQ, and PNH were newly developed from their operational definitions and the characteristics of AI-generated short-video experiences. PCO items were adapted from the information-overload dimension of the Technology Overload Scale [34]. AIF items were adapted from the cognitive and emotional dimensions of the Social Media Fatigue Scale [13]. SAT and CVI items were adapted from satisfaction and continuance-intention measures based on the expectation-confirmation model [35].**

## **Before formal data collection, five experts independently reviewed the candidate items in one review round. Their backgrounds covered media psychology, communication and human–computer interaction, psychometrics and statistics, survey methodology, and short-video industry or content governance. The review focused on relevance, clarity, comprehensibility for Chinese short-video users, and contextual appropriateness.**

## **Based on the consolidated written feedback, several items were revised to clarify construct boundaries and avoid ambiguous or potentially causal wording. PLQ8 was removed before formal administration because it assessed perceived informational value rather than the audiovisual, logical, and production-quality characteristics defined by PLQ. The final questionnaire retained PLQ1–PLQ7. No separate participant pilot study or formal cognitive interview was conducted. The complete item-source, revision, deletion, and retention information is provided in Supplementary Table S1.**

## **Comment 3**

## **Response: Thank you for identifying this inconsistency. PLQ8 was an initial candidate item in the questionnaire-development stage but was removed after expert content review and before formal data collection. The item assessed perceived informational value rather than the audiovisual, logical, and production-quality characteristics defined by PLQ. Therefore, the deletion was theory- and content-based rather than data-driven.**

## **PLQ8 was not included in the final administered questionnaire, the analytic dataset, or any statistical analysis. The final PLQ measure therefore consisted of PLQ1–PLQ7. Because PLQ8 was removed before data collection and no responses to this item were available, no statistical robustness analysis involving PLQ8 was applicable. The deletion decision was made before inspecting formal survey responses and is now clarified in the Methods and Supplementary Table S1.**

## **Comment 4**

## **Response: Thank you for raising this important concern. We agree that some PCO and AIF items refer to related experiences involving attention and information processing. We have clarified their conceptual distinction: PCO represents a resource-oriented appraisal of the perceived demands involved in identifying, filtering, and evaluating AI-generated videos, whereas AIF represents a subsequent fatigue state involving cognitive exhaustion, irritation, boredom, and reduced viewing interest.**

## **Empirically, the latent correlation between PCO and AIF was .612, and their HTMT value was .608. In addition, the two-factor model fit substantially better than the one-factor model, with Δχ²(6) = 518.186, p < .001, and ΔCFI = .057. These results support their empirical distinction. However, we acknowledge that semantic similarity in references to attention and processing may have increased their observed association. We have added this possibility to the Limitations section and now interpret the discriminant-validity evidence cautiously.**

## **We again thank the Academic Editor and the two reviewers for their time and constructive guidance. We appreciate the opportunity to revise the manuscript and hope that the revised version is now suitable for further consideration.**

## **Sincerely,**

## **The Authors**
