# Supplementary material for: Perceived AIGC saturation and AI content fatigue on short-video platforms: An appraisal-based structural equation modeling study
Source: PLoS One. 2026 Oct 16;21(10):e0360731. doi: 10.1371/journal.pone.0360731 (PMC13674767; doi:10.1371/journal.pone.0360731)
Supplement: Peer review history

# Peer review: Decision Letter 0

**DOI:** 10.1371/journal.pone.0360731.r001

**Authors:** Donghwa Chung

31 Aug 2026

Dear Dr. Tang,

Thank you for submitting your manuscript to PLOS One. After careful consideration, we feel that it has merit but does not fully meet PLOS One’s publication criteria as it currently stands. Therefore, we invite you to submit a revised version of the manuscript that addresses the points raised during the review process.

==============================

I sincerely appreciate the reviewers’ time and effort in carefully reviewing the manuscript and providing constructive comments, despite their busy schedules.

After carefully considering both sets of comments, several issues require further attention.

The measurement information should be clarified thoroughly, particularly whether each measure was newly developed for this study or derived/adapted from previous studies. As Reviewer 1 noted, some validity analysis results appear to be missing, and the model-fit indices seem to be duplicated between the CFA and hypothesized model. Please recheck the analyses and report the exact statistical results throughout the manuscript.

The ethical approval information also needs to be updated. Please provide the full name of the ethics committee and the exact approval date.

I also agree with Reviewer 2 that the conceptualization of the core constructs is not sufficiently consistent throughout the manuscript. These definitions and conceptualizations should be revised to ensure consistency.

Please address the remaining comments from both reviewers point by point in the revised manuscript and response letter.

==============================

Please submit your revised manuscript by Oct 30 2026 11:59PM. If you will need more time than this to complete your revisions, please reply to this message or contact the journal office at plosone@plos.org. When you're ready to submit your revision, log on to https://www.editorialmanager.com/pone/ and select the 'Submissions Needing Revision' folder to locate your manuscript file.

Please include the following items when submitting your revised manuscript:

- A letter that responds to each point raised by the academic editor and reviewer(s). You should upload this letter as a separate file labeled 'Response to Reviewers'.
- A marked-up copy of your manuscript that highlights changes made to the original version. You should upload this as a separate file labeled 'Revised Manuscript with Track Changes'.
- An unmarked version of your revised paper without tracked changes. You should upload this as a separate file labeled 'Manuscript'.

If you would like to make changes to your financial disclosure, please include your updated statement in your cover letter. Guidelines for resubmitting your figure files are available below the reviewer comments at the end of this letter.

If applicable, we recommend that you deposit your laboratory protocols in protocols.io to enhance the reproducibility of your results. Protocols.io assigns your protocol its own identifier (DOI) so that it can be cited independently in the future. For instructions see: https://journals.plos.org/plosone/s/submission-guidelines#loc-laboratory-protocols. Additionally, PLOS One offers an option for publishing peer-reviewed Lab Protocol articles, which describe protocols hosted on protocols.io. Read more information on sharing protocols at https://plos.org/protocols?utm_medium=editorial-email&utm_source=authorletters&utm_campaign=protocols.

As the corresponding author, your ORCID iD is verified in the submission system and will appear in the published article. PLOS supports the use of ORCID, and we encourage all coauthors to register for an ORCID iD and use it as well. Please encourage your coauthors to verify their ORCID iD within the submission system before final acceptance, as unverified ORCID iDs will not appear in the published article. *Only*  the individual author can complete the verification step; PLOS staff *cannot*  verify ORCID iDs on behalf of authors.

We look forward to receiving your revised manuscript.

Kind regards,

Donghwa Chung

Academic Editor

PLOS One

Journal Requirements:

When submitting your revision, we need you to address these additional requirements.

1.Please ensure that your manuscript meets PLOS ONE's style requirements, including those for file naming. The PLOS ONE style templates can be found at

https://journals.plos.org/plosone/s/file?id=wjVg/PLOSOne_formatting_sample_main_body.pdf and

https://journals.plos.org/plosone/s/file?id=ba62/PLOSOne_formatting_sample_title_authors_affiliations.pdf

2. We note that Figure 2 in your submission contain copyrighted images. All PLOS content is published under the Creative Commons Attribution License (CC BY 4.0), which means that the manuscript, images, and Supporting Information files will be freely available online, and any third party is permitted to access, download, copy, distribute, and use these materials in any way, even commercially, with proper attribution. For more information, see our copyright guidelines: http://journals.plos.org/plosone/s/licenses-and-copyright.

We require you to either (1) present written permission from the copyright holder to publish these figures specifically under the CC BY 4.0 license, or (2) remove the figures from your submission:

1. You may seek permission from the original copyright holder of Figure 2  to publish the content specifically under the CC BY 4.0 license.

We recommend that you contact the original copyright holder with the Content Permission Form (http://journals.plos.org/plosone/s/file?id=7c09/content-permission-form.pdf) and the following text:

“I request permission for the open-access journal PLOS ONE to publish XXX under the Creative Commons Attribution License (CCAL) CC BY 4.0 (http://creativecommons.org/licenses/by/4.0/). Please be aware that this license allows unrestricted use and distribution, even commercially, by third parties. Please reply and provide explicit written permission to publish XXX under a CC BY license and complete the attached form.”

Please upload the completed Content Permission Form or other proof of granted permissions as an "Other" file with your submission.

In the figure caption of the copyrighted figure, please include the following text: “Reprinted from [ref] under a CC BY license, with permission from [name of publisher], original copyright [original copyright year].”

2. If you are unable to obtain permission from the original copyright holder to publish these figures under the CC BY 4.0 license or if the copyright holder’s requirements are incompatible with the CC BY 4.0 license, please either i) remove the figure or ii) supply a replacement figure that complies with the CC BY 4.0 license. Please check copyright information on all replacement figures and update the figure caption with source information. If applicable, please specify in the figure caption text when a figure is similar but not identical to the original image and is therefore for illustrative purposes only.

3. If the reviewer comments include a recommendation to cite specific previously published works, please review and evaluate these publications to determine whether they are relevant and should be cited. There is no requirement to cite these works unless the editor has indicated otherwise.

Additional Editor Comments:

I appreciate the reviewers’ time and effort in carefully reviewing the manuscript and providing constructive comments for further revision, despite their busy schedules.

After carefully reviewing both sets of comments, several issues require further attention.

The measurement information should be clarified thoroughly, particularly whether each measure was newly developed for this study or derived/adapted from previous studies. As Reviewer 1 noted, some validity analysis results appear to be missing, and the reported model-fit indices seem to be duplicated between the CFA and hypothesized model. Please carefully recheck the analyses and report the exact statistical results throughout the manuscript. The ethical approval information also needs to be updated. Please provide the full name of the ethics committee and the exact approval date.

I also agree with Reviewer 2 that the conceptualization of the core constructs is not sufficiently consistent throughout the manuscript. These definitions and conceptualizations should be revised to ensure consistency.

Please address the remaining comments from both reviewers point by point in the revised manuscript and response letter.

Sincerely,

Donghwa Chung

[Note: HTML markup is below. Please do not edit.]

Reviewer's Responses to Questions

**Comments to the Author**

1. Is the manuscript technically sound, and do the data support the conclusions?

Reviewer #1: Partly

Reviewer #2: Yes

**********

2. Has the statistical analysis been performed appropriately and rigorously?

Reviewer #1: No

Reviewer #2: Yes

**********

3. Have the authors made all data underlying the findings in their manuscript fully available?

The PLOS Data policy

Reviewer #1: No

Reviewer #2: Yes

**********

4. Is the manuscript presented in an intelligible fashion and written in standard English?

Reviewer #1: Yes

Reviewer #2: Yes

**********

Reviewer #1: I thank the authors for the interesting read. The manuscript addresses a timely topic and has potential value. However, major revisions are required to establish the validity, transparency, and reproducibility of the findings.

Major Comments

(1) The measure-development process is insufficiently reported. The manuscript only states that the questionnaire was developed from prior literature, expert feedback, and iterative refinement. Please report the item sources, item-generation process, number and expertise of reviewers, evaluation criteria, pilot or cognitive testing, survey language, translation procedures if applicable, and item-retention decisions.

(2) The claim that the questionnaire was “psychometrically developed” is stronger than the evidence reported. The same sample of 513 respondents was used for the CFA and structural analysis, and no exploratory analysis or independent validation sample is described. Please clarify whether the factor structure was specified fully in advance and provide any prior pilot or development-sample evidence. Otherwise, describe the present validation as preliminary and acknowledge the absence of independent confirmation.

(3) Discriminant validity is incompletely reported. The manuscript states only that all HTMT values were below .90 and that the maximum was .878. Please provide the full latent-correlation and HTMT matrices and report the values specifically involving perceived content overload, AI content fatigue, and general short-video fatigue. Targeted one-factor versus two-factor comparisons would strengthen the evidence that these closely related constructs are distinct.

(4) The distinction between AI content fatigue and general short-video fatigue is not demonstrated in the reported results. The Methods state that the two constructs were modelled as distinct, but general short-video fatigue is absent from Table 1, Fig 2, and the structural results. Please report its items, reliability, factor loadings, correlations, discriminant-validity results, and whether AI content fatigue predicts satisfaction and continued viewing intention beyond general short-video fatigue.

(5) The CFA and structural model are reported with exactly the same fit statistics and degrees of freedom. Both are reported as χ²(426) = 1007.883, CFI = .935, TLI = .929, IFI = .935, and RMSEA = .052. This suggests that the structural-model output may have been repeated in the CFA section or that the stated “measurement model” was not a freely correlated CFA. Please verify the analyses and report separate model specifications, degrees of freedom, parameter counts, fit indices, and software output for the CFA and SEM.

(6) Essential analytical details are missing. Please identify the statistical software and version, SEM estimator, treatment of the seven-point items as continuous or ordinal, missing-data procedure, assessment of non-normality, use of robust standard errors or scaled fit indices, factor-scaling method, bootstrap procedure, and any correlated residuals or modification-index-driven changes. Please also report SRMR.

(7) The stated covariates do not appear in the reported structural model. The manuscript says that general short-video fatigue, negative AI attitudes, AI-identification confidence, exposure frequency, platform-use intensity, active following of AI accounts, and demographic variables were controlled, but they are absent from Fig 2 and Tables 2–3. Moreover, several R² values exactly match squared single-predictor coefficients—for example, .581² ≈ .338, .566² ≈ .320, and .578² ≈ .334—indicating that the reported model likely did not include the stated covariates. Please report clearly labelled unadjusted and adjusted models, all covariate paths and coefficients, and the correct adjusted R² values.

(8) The reported indirect effects do not fully correspond to the stated hypotheses. H5 and H6 specify paths beginning with perceived AIGC saturation, such as PAS → PLQ → AIF → SAT. Table 4 instead reports several shorter paths beginning with PLQ, PCO, or PNH. Please report every hypothesised pathway—H4a–c, H5a–c, and H6a–c—separately, with unstandardised and standardised estimates, bootstrap standard errors, and bias-corrected 95% confidence intervals. A significant aggregate indirect effect does not establish support for every component pathway.

(9) The model constrains all direct effects from perceived AIGC saturation to zero. This imposes complete mediation rather than comparing complete and partial mediation. Please justify these constraints theoretically and test a sensitivity model containing direct paths from perceived saturation to AI content fatigue, satisfaction, and continued viewing intention.

(10) The inference for narrative homogenisation is inconsistent. The PNH → AIF path has an ML p-value of .010 but a bootstrap p-value of .051, while one indirect effect has a confidence interval ending at .000 but is described as significant at p = .048. Please specify the primary inferential criterion, report additional decimal places, and base conclusions consistently on the bootstrap confidence intervals. A result that includes zero should not be described as supported or “marginally robust.”

(11) The data-screening description is internally inconsistent. The Methods state that participants were excluded for failing both attention checks and several other criteria, whereas the Results state that all 23 exclusions involved failing at least one attention check. Please reconcile these statements and provide a participant-flow table showing the number excluded under each criterion and any overlap. Summarise the exact completion-time, long-string, contradiction, duplicate, and attention-check thresholds in the Methods and cross-reference S3. Also clarify whether “pre-specified” means that the rules were established before examining the responses.

(12) The theoretical meaning of “appraisal mechanisms” requires clarification. Perceived low-quality prevalence and narrative homogenisation appear to be perceived content characteristics, whereas content overload is closer to a cognitive response. Please explain how each construct maps onto Cognitive Appraisal Theory and the S–O–R framework and why these three mechanisms were selected over alternatives such as perceived authenticity, deception, trust, novelty loss, or algorithmic intrusiveness.

(13) The ethics information is insufficiently specific. “The Academic Committee of the author’s institution” does not identify the institution, committee, or its authority to issue a research-ethics exemption. Please provide the full committee and institution names, the exemption or review reference number if issued, and clarify that consent was recorded electronically. If no reference number was issued, state this explicitly.

(14) The data-availability statements are inconsistent. The Methods state that the materials “will be made available” upon publication, whereas the Data Availability Statement says that they “are available” on Zenodo. Please reconcile these statements and ensure that the record is accessible to reviewers. S1–S4 are listed in the manuscript, so the issue is not necessarily their absence; rather, the authors should ensure that the files and repository permit full reproduction. In particular, provide executable analysis syntax or code in addition to statistical output.

(15) The definition of AI-generated content is broad and heterogeneous. Content that is fully generated and content that is only “substantially assisted” by AI may produce very different user responses. Please clarify how participants distinguished these categories, whether examples were provided, and how possible misclassification affects the interpretation of perceived saturation.

Minor Comments

(16) Some discussion statements remain too causal for cross-sectional data. Although the manuscript acknowledges this limitation, it states that fatigue “emerges” from appraisals, that saturation “triggers” appraisal processes, and that certain appraisals “drive” fatigue. Please use associational language consistently and acknowledge that fatigue or dissatisfaction could also influence negative appraisals of AIGC.

(17) The sample is inadequately described. Please provide a complete sample-characteristics table reporting age, gender, education, residential area, principal platform, exposure frequency, use intensity, AI-identification confidence, active following or searching of AI-content accounts, recruitment source, and participant compensation, if any. Please also report how many individuals were screened out before entering the main questionnaire. The requirement that participants recognise “reasonably identifiable” AIGC may select users with unusually high AI awareness or exposure and should be discussed as a source of selection bias.

(18) No sample-size rationale is reported. Please state whether the target sample was determined prospectively and provide an appropriate model-based justification. If no prospective calculation was conducted, state this transparently and provide a sensitivity or precision analysis rather than presenting a retrospective calculation as an a priori analysis.

(19) The common-method-bias conclusion is too strong. Harman’s single-factor test cannot establish that common-method variance did not materially influence the findings. Please describe the test as a limited diagnostic and more clearly acknowledge that all focal variables were collected from the same respondents, at the same time, using similar response scales and similarly valenced items.

(20) The exclusively negative model does not fully support some of the manuscript’s broader conclusions. All three appraisal variables are negatively valenced, which may contribute to shared-valence associations with fatigue and dissatisfaction. The model therefore cannot establish that users do not react negatively to AIGC itself or that only negative appraisals matter. Please temper these conclusions and acknowledge that positive or neutral appraisals were not measured.

(21) Claims that low-quality prevalence was the “strongest” predictor are not formally tested. Numerically different standardised coefficients do not establish that the coefficients differ significantly. Please conduct a Wald or bootstrap comparison of coefficients or revise the wording to “had the largest estimated coefficient.”

(22) Several practical recommendations exceed the evidence. The study did not test granular AIGC labels, recommendation controls, automated duplicate detection, quality-sensitive moderation, or prioritisation of human-created content. Please present these as tentative design proposals requiring experimental evaluation rather than as interventions demonstrated by the present findings.

(23) The manuscript would benefit from engaging with two recent studies that are directly relevant to its theoretical framework. The study on AI fatigue (https://doi.org/10.1016/j.chbr.2026.101186) offers a validated conceptualisation and measure of fatigue arising from human–AI interaction and can help strengthen the definition of AI content fatigue and clarify its distinction from related fatigue constructs. The study on critical thinking in AI use (https://doi.org/10.1016/j.chbr.2026.101103) identifies verification, motivation, and reflection as important dimensions of how users evaluate AI-generated information and can enrich the discussion of the cognitive effort involved in identifying, filtering, and assessing AIGC. Engaging with these studies would strengthen the manuscript’s theoretical positioning and clarify how the present constructs extend existing research.

(24) The weak narrative-homogenisation result is overinterpreted. The manuscript calls it “theoretically meaningful rather than a mere statistical artifact,” although the bootstrap result does not meet the conventional .05 threshold. Please interpret this finding cautiously and acknowledge possible measurement overlap, suppression among correlated appraisal variables, or sampling uncertainty.

Reviewer #2: The manuscript addresses a timely topic and proposes a potentially useful framework for understanding AI content fatigue on short-video platforms. The study is generally readable, but several issues concerning conceptual organization, construct development, measurement transparency, and questionnaire design should be addressed to strengthen the rigor and clarity of the manuscript.

1. The manuscript should be substantially streamlined. Several core arguments and construct definitions are repeated across the Introduction, hypothesis-development sections, Measures, Discussion, and Conclusion. In particular, the three appraisal constructs are defined multiple times. I suggest defining each construct concisely in the Introduction, focusing the hypothesis-development sections on theoretical relationships, and reserving the Measures section for operationalization. This would improve concision and strengthen the progression from conceptual definition to theoretical prediction and empirical measurement.

2. The development of the AIGC-specific measures requires greater transparency. Several central constructs appear to be newly developed or substantially contextualized for AIGC short-video environments. The manuscript should provide more detail on item generation, theoretical sources, the number and expertise of reviewers, criteria for item revision or deletion, whether pilot testing or cognitive interviews were conducted, and how the final items were selected. Such information is important for evaluating the content validity of these measures.

3. The number of PLQ items is inconsistent between the questionnaire and the manuscript. The administered questionnaire contains eight perceived low-quality prevalence items (Items 5–12), whereas Table 1 reports PLQ as a seven-item construct. The authors should identify the deleted item, explain the theoretical or statistical basis for its removal, and clarify whether the deletion criteria were specified in advance. If the deletion was data-driven, this process should be reported transparently and supported by an appropriate robustness analysis.

4. Potential conceptual overlap between content overload and AI content fatigue should be examined more carefully. Several content-overload items refer directly to “heavy burden,” excessive attentional demands, and difficulty processing information, while AI-fatigue items assess attentional difficulty and cognitive exhaustion. This semantic proximity may partly inflate the PCO–AIF relationship. The authors should provide stronger evidence that these constructs are conceptually and empirically distinct and discuss whether item wording contributes to their observed association.

**********

what does this mean?). If published, this will include your full peer review and any attached files.

If you choose “no”, your identity will remain anonymous but your review may still be made public.

**Do you want your identity to be public for this peer review?**  For information about this choice, including consent withdrawal, please see our Privacy Policy

Reviewer #1: No

Reviewer #2: No

**********

[NOTE: If reviewer comments were submitted as an attachment file, they will be attached to this email and accessible via the submission site. Please log into your account, locate the manuscript record, and check for the action link "View Attachments". If this link does not appear, there are no attachment files.]

To ensure your figures meet our technical requirements, please review our figure guidelines: https://journals.plos.org/plosone/s/figures

You may also use PLOS’s free figure tool, NAAS, to help you prepare publication quality figures: https://journals.plos.org/plosone/s/figures#loc-tools-for-figure-preparation.

NAAS will assess whether your figures meet our technical requirements by comparing each figure against our figure specifications.

# Author comment: Author response to Decision Letter 1

**DOI:** 10.1371/journal.pone.0360731.r002

5 Sep 2026

Dear Dr. Donghwa Chung and Reviewers,

Thank you for the careful editorial assessment and constructive comments from the Academic Editor and the two anonymous reviewers. We have revised the manuscript point by point and addressed all comments in detail.

The revisions include clarification of the questionnaire-development process, item sources and retention decisions, the preliminary status of the CFA, discriminant-validity evidence, GSVF measurement and control analyses, separate CFA and SEM fit statistics, adjusted structural paths and R² values, bootstrap indirect effects, sample-screening procedures, ethics information, AIGC definitions, common-method-bias limitations, sample-size limitations, and the interpretation of non-significant paths.

We have also updated Figures 1 and 2, Supplementary Tables S1–S10, the cleaned analysis dataset, the codebook, the AMOS files, the R analysis scripts, and the Zenodo records. All changes in the revised manuscript are marked. A detailed point-by-point response to the Academic Editor, Reviewer 1, and Reviewer 2 follows below.

Reviewer 1

Comment 1

Response: Thank you for identifying this important omission. We agree that the original manuscript did not sufficiently describe the questionnaire-development process. We have therefore expanded the Methods section to clarify the sources and generation of each construct’s items, the language and adaptation procedures, the expert-review process, the evaluation dimensions, and the item-retention decisions. The questionnaire was administered in Simplified Chinese. PAS, PLQ, and PNH were newly developed from their operational definitions and the AIGC short-video context. PCO items were adapted from the information-overload dimension of the Technology Overload Scale, AIF items were adapted from the cognitive and emotional dimensions of the Social Media Fatigue Scale, and SAT and CVI items were adapted from satisfaction and continuance-intention measures based on the expectation-confirmation model. Before formal data collection, five experts with backgrounds in media psychology, communication and human–computer interaction, psychometrics and statistics, survey methodology, and short-video industry/content governance independently reviewed the items in one review round. They evaluated relevance, clarity, comprehensibility, and contextual appropriateness. The research team then consolidated the feedback and implemented the wording revisions. No separate participant pilot study or formal cognitive interview was conducted. PLQ8 was removed before formal administration because it assessed perceived informational value rather than the audiovisual, logical, and production-quality aspects defined by the PLQ construct. The final administered questionnaire therefore contained PLQ1–PLQ7. The item sources, wording changes, and retention decisions are now documented in the revised Methods section and Supplementary Table S1. The final questionnaire and coding information are also provided in the supplementary materials.

Comment 2

Response: Thank you for raising this important concern. We agree that the phrase “psychometrically developed questionnaire” overstated the level of evidence provided by the present study. The seven-factor measurement structure for the core constructs—PAS, PLQ, PNH, PCO, AIF, SAT, and CVI—was specified before formal data collection on the basis of the construct definitions, item-generation rationale, and expert review. However, the factor structure was not developed through an exploratory factor analysis in the present dataset, and no separate pilot, development sample, or independent CFA sample was available. All 513 valid responses were used for the CFA and subsequent SEM analyses. We have therefore replaced “psychometrically developed questionnaire” with “theory-informed, context-specific questionnaire” and now describe the CFA results as preliminary measurement evidence rather than independent scale validation. We have also added a limitation stating that the measurement structure requires replication using an independent sample and, ideally, a separate EFA–CFA sequence. PLQ8 was removed before formal administration on conceptual grounds following expert review; no item was deleted after inspecting the CFA results from the 513 respondents.

Comment 3

Response: Thank you for this helpful suggestion. We agree that the original manuscript provided only a summary statement about HTMT values and did not show the complete discriminant-validity evidence. We have now added the complete latent-correlation matrix and HTMT matrix to the Supplementary Materials. In the seven-factor CFA, the largest latent correlation was between SAT and CVI (r = .879). The latent correlation between PCO and AIF was r = .612. In the supplementary model including general short-video fatigue (GSVF), the latent correlations were r = .742 for AIF–GSVF and r = .431 for PCO–GSVF. The corresponding HTMT values were .608, .741, and .429, respectively. All HTMT values were below .90. We also conducted nested one-factor versus two-factor CFA comparisons. For PCO and AIF, the two-factor model fit substantially better than the one-factor model, Δχ²(6) = 518.186, p < .001, ΔCFI = .057. For AIF and GSVF, the two-factor model also fit substantially better, Δχ²(7) = 503.573, p < .001, ΔCFI = .046. These results provide additional evidence that PCO, AIF, and GSVF represent empirically distinguishable constructs, although their conceptual relatedness is acknowledged.

Comment 4

Response: Thank you for pointing out this omission. We agree that the original manuscript mentioned general short-video fatigue (GSVF) as a control construct but did not report its measurement properties or its role in the structural model. We have now added the complete GSVF item wording and coding information to the Supplementary Materials. GSVF was measured using six context-adapted items covering attention difficulty, cognitive tiredness, mental fatigue, boredom, irritation, and reduced viewing interest during general short-video use. The six GSVF items showed standardized loadings ranging from .753 to .783. Internal consistency was satisfactory (Cronbach’s α = .895; McDonald’s ω = .895), with composite reliability of .895 and AVE of .586. The latent correlation between GSVF and AIF was r = .742, and their HTMT value was .741. The two-factor model distinguishing AIF from GSVF fit substantially better than the one-factor model. After controlling for GSVF and the other covariates, AIF remained negatively associated with satisfaction (β = −.575, p < .001) and continued viewing intention (β = −.629, p < .001). These findings indicate that AI content fatigue retains explanatory value beyond general short-video fatigue.

Comment 5

Response: Thank you for identifying this important reporting problem. We re-checked the archived AMOS outputs and confirmed that the original manuscript inadvertently reported the same fit statistics for the CFA and structural model. We have corrected this error and now report the two models separately. The measurement model was a freely correlated seven-factor CFA including PAS, PLQ, PNH, PCO, AIF, SAT, and CVI. It contained 83 estimated parameters and 413 degrees of freedom. The adjusted structural model included the measurement component, structural paths, GSVF, AIATTneg, exposure and usage controls, and demographic covariates. It contained 449 estimated parameters and 1,321 degrees of freedom. The revised manuscript now reports the model specifications, parameter counts, degrees of freedom, and fit indices separately. The corresponding AMOS output files are provided in the supplementary analysis materials.

Comment 6

Response: Thank you for requesting these methodological details. We have expanded the Statistical analysis section to clarify the software, estimator, treatment of the Likert-scale items, missing-data handling, distributional checks, factor scaling, bootstrap procedure, residual specification, and model-modification procedure. The CFA and SEM analyses were conducted using IBM SPSS Amos26 with maximum likelihood estimation. The seven-point Likert items were treated as approximately continuous variables because they had seven response categories and showed limited skewness and kurtosis. Item-level skewness ranged from −.177 to .188, and excess kurtosis ranged from −.425 to .323. No missing core-item values were present in the final analytic sample, and no imputation was performed. The first loading of each latent factor was fixed to 1.0 for identification. No correlated indicator residuals were specified, and no post hoc structural paths or residual covariances were added on the basis of modification indices. Robust standard errors and scaled fit indices were not used in the AMOS maximum-likelihood analyses. Indirect effects were evaluated using 5,000 usable bootstrap samples and bias-corrected 95% confidence intervals. The revised manuscript now reports SRMR separately from AMOS’s RMR. The AMOS output for the adjusted structural model showed a standardized RMR of .0696. The SRMR for the matched supplementary lavaan CFA was .028.

Comment 7

Response: Thank you for identifying this inconsistency. We agree that the original manuscript did not clearly distinguish the unadjusted and covariate-adjusted structural models, and that the presentation of the R² values could suggest that the covariates had not been included.

We have now estimated and reported two structural specifications. The unadjusted model included only the hypothesized paths among PAS, PLQ, PNH, PCO, AIF, SAT, and CVI. The covariate-adjusted model additionally included GSVF, AIATTneg, CONF, EXPOSURE, USE, ACTIVE, and dummy-coded age, gender, education, and residential-area variables. Each covariate was specified as an observed exogenous predictor of AIF, SAT, and CVI. The covariate-adjusted model is treated as the primary structural model.

The unadjusted model had χ²(426) = 1007.883, CFI = .935, TLI = .929, and RMSEA = .052. The adjusted model had χ²(1321) = 1991.541, CFI = .946, TLI = .929, RMSEA = .031, and standardized RMR = .0696.

In the adjusted model, the R² values were .364 for PLQ, .288 for PNH, .216 for PCO, .747 for AIF, .351 for SAT, and .374 for CVI. These values are the squared multiple correlations obtained from the covariate-adjusted model.

The complete coefficients for the hypothesized paths and all covariate paths are now reported in Table 2 and Supplementary Table S6. Figure 2 has been revised to display the focal structural paths and the GSVF control paths. The remaining covariate paths are reported in full in Supplementary Table S6 and were omitted from the figure for visual clarity. The previously reported coefficients and R² values are now explicitly labelled as results from the unadjusted model.

Comment 8

Response: Thank you for identifying this inconsistency. We agree that the previous Table 4 mixed path-specific indirect effects with shorter component-specific indirect paths. We have now reorganized the indirect-effect analysis according to the prespecified hypotheses.

H4a–H4c represent PAS → appraisal mechanism → AIF. H5a–H5c represent PAS → appraisal mechanism → AIF → SAT. H6a–H6c represent PAS → appraisal mechanism → AIF → CVI. For each pathway, we now report the unstandardized estimate, standardized estimate, bootstrap SE, bias-corrected 95% confidence interval, and bootstrap p value.

The revised results show that the indirect pathways through PLQ and PCO were supported, whereas the corresponding pathways through PNH were not supported. We also clarify that significance of an overall indirect effect does not establish significance of every individual component pathway.

Comment 9

Response: Thank you for raising this important point. We agree that the original manuscript did not sufficiently explain why the primary model omitted direct paths from PAS to AIF, SAT, and CVI.

The primary model was specified as an indirect-only model because the theoretical framework focuses on appraisal-based mechanisms. Specifically, perceived AIGC saturation was expected to be associated with downstream outcomes through perceived low-quality prevalence, perceived narrative homogenization, perceived content overload, and AI content fatigue. The omission of direct paths was therefore theory-driven and was not intended to imply that direct associations were impossible.

To assess the robustness of this specification, we estimated a sensitivity model using R/lavaan by adding direct paths from PAS to AIF, SAT, and CVI while retaining the hypothesized paths and covariates. Both the sensitivity model and its comparison model used the same 513 respondents, variables, estimator, and model parameterization. The additional paths were not significant, and the matched R/lavaan model comparison showed no material improvement in fit, Δχ²(3) = .937, p = .816. This comparison was based solely on the two matched R/lavaan models and was not calculated from the AMOS fit statistics for the primary adjusted structural model. The complete sensitivity results are reported in Supplementary Table S7.

We have clarified that the sensitivity results are consistent with the indirect-only specification, but do not provide definitive proof of complete mediation.

Comment 10

Response: Thank you for identifying this inconsistency. We agree that the previous manuscript mixed maximum-likelihood p values, bootstrap p values, and rounded confidence intervals when interpreting the narrative-homogenization pathway.

We have now specified the bias-corrected 95% bootstrap confidence interval as the primary inferential criterion for indirect effects. A pathway is considered supported only when its confidence interval excludes zero. ML p values are reported as supplementary information and are not used to override the bootstrap-based conclusion.

In the covariate-adjusted model, the PNH → AIF path was β = .036, ML p = .298, bootstrap p = .467, with a bias-corrected 95% CI of [−.052, .116]. Therefore, this path is classified as not supported.

We also replaced rounded confidence limits and report the relevant estimates to at least three decimal places. Any confidence interval that includes zero is now described as not supported, regardless of whether a separate p value is below .05.

Comment 11

Response: Thank you for identifying this inconsistency. We have revised the Methods and Results sections to distinguish prespecified exclusion rules from supplementary data-quality checks.

Of the 536 initial responses, seven failed AC1 only and 16 failed AC2 only. No respondent failed both attention checks. Thus, 23 responses were excluded for failing at least one attention check, and 513 valid responses remained. No additional cases were excluded because of missing core items, completion time, long-string responding, contradictory responses, or duplicate records.

We have added a participant-flow table and reported the completion-time distribution for the final valid sample. The detailed variable coding and screening rules are cross-referenced in Supplementary File S3.

Comment 12

Response: Thank you for this important theoretical clarification. We have revised the manuscript to explain how the three appraisal mechanisms map onto Cognitive Appraisal Theory and the S–O–R framework.

PLQ and PNH are conceptualized as content-focused subjective appraisals of perceived production quality and narrative similarity. PCO is conceptualized as a resource-oriented appraisal of the cognitive demands required to identify, filter, and evaluate AI-generated videos. It does not measure fatigue itself. In the S–O–R model, perceived AIGC saturation functions as the stimulus, PLQ, PNH, and PCO represent organism-level appraisal processes, AI content fatigue represents a subsequent internal state, and satisfaction and continued viewing intention represent downstream responses.

We also clarify that these three mechanisms were selected to represent production-quality, narrative-similarity, and cognitive-processing dimensions of AI short-video experience. Perceived authenticity, deception, trust, novelty loss, and algorithmic intrusiveness are plausible alternative mechanisms, but they were not measured in this study. We now acknowledge this limitation and identify these constructs as directions for future research.

Comment 13

Response: Thank you for this comment. We have revised the ethics statement to identify the institution and reviewing committee. The protocol was reviewed by the Academic Committee of Jinling Institute of Technology, which confirmed that the project met the institutional criteria for exemption from full ethical review. The exemption statement was issued on May 13, 2026. No separate exemption or approval number was provided by the committee. We have also clarified that informed consent was recorded electronically through a required consent item before participants could access the questionnaire.

Comment 14

Response: Thank you for identifying this inconsistency. We have aligned the Data Availability Statement with the Zenodo record and identified the de-identified dataset, questionnaire, codebook, and supplementary tables deposited there. The AMOS model files, AMOS output files, executable R scripts, and README are included in the companion Zenodo record. in the same record before resubmission and will be included in the final repository description once the updated record is published. These materials are intended to support reproduction of the descriptive statistics, CFA, reliability and validity analyses, adjusted SEM, bootstrap indirect effects, and sensitivity analyses.

Comment 15

Response: Thank you for raising this important issue. We have clarified that the study used an umbrella definition of AIGC. Fully generated videos were defined as videos in which major visual, audio, animation, character, or script elements were generated by generative AI. Substantially AI-assisted videos were defined as videos in which generative AI materially contributed to one or more major audiovisual or script elements.

Participants were not required to classify each video according to the degree of AI involvement. Both fully generated and substantially AI-assisted videos were included when participants could reasonably identify them as AI-related. The questionnaire did not provide standardized video examples; instead, it provided written identification cues, including platform labels, creator disclosures, account information, and visible generative features.

Accordingly, perceived AIGC saturation should be interpreted as perceived saturation of identifiable AI-generated or substantially AI-assisted short videos, rather than as an estimate of the objective prevalence of fully AI-generated content. Potential misclassification may have introduced measurement error. If misclassification was nondifferential, the observed associations may have been attenuated; if it was related to AI attitudes or identification confidence, bias in either direction is possible.

Comment 16

Response: Thank you for this important comment. We agree that several sentences in the Discussion used stronger causal language than warranted by the cross-sectional design. We have replaced terms such as “emerges,” “triggers,” “drives,” and “produces” with association-based wording. We also clarified that the observed direction is theoretically specified but cannot establish temporal ordering or causality. In addition, we acknowledged that fatigue or dissatisfaction may have influenced participants’ negative evaluations of AI-generated content and their reports of perceived AIGC saturation.

Comment 17

Response: Thank you for this suggestion. We have added a complete sample-characteristics table reporting age, gender, education, residential area, most frequently used platform, AI-content exposure frequency, daily short-video use intensity, AI-identification confidence, and active following or searching for AI-content accounts. We have also clarified the recruitment channels, compensation arrangements, and the distinction between eligibility screening before the main questionnaire and post-completion data-quality exclusions.

Of the 536 completed responses, 23 were excluded after completion because they failed at least one attention check, leaving 513 valid responses. The eligibility requirement concerning reasonably identifiable AI-generated content may have preferentially selected participants with greater AI awareness or exposure. We have added this possibility as a potential source of selection bias in the Limitations section.

Comment 18

Response: Thank you for this comment. No formal a priori power calculation or preregistered sample-size target was conducted before data collection. The final sample size was determined by the feasible recruitment of eligible participants. We have removed any wording that could imply that the sample was prospectively powered. Instead, we now describe the complexity of the measurement and structural models and provide a confidence-interval-based precision assessment. These results are presented as preliminary evidence rather than as confirmation of an a priori power calculation.

Comment 19

Response: Thank you for this important comment. We agree that Harman’s single-factor test cannot establish that common-method variance had no substantive influence on the findings. We have therefore revised the manuscript to describe Harman’s test only as a limited supplementary diagnostic.

We now explicitly acknowledge that all core constructs were measured from the same 513 respondents at a single time point, using similarly structured seven-point self-report scales. Several constructs also involved related evaluative or affective wording, which may increase the risk of shared method variance. Although the first unrotated factor accounted for 37.67% of the variance, this result is not interpreted as evidence that common-method bias was absent. The manuscript now states that common-method variance cannot be ruled out and that the findings should be interpreted with this limitation in mind.

Comment 20

Response: Thank you for this important observation. We agree that the three appraisal constructs primarily capture negative evaluations of AI-generated content, which may share evaluative valence with AI content fatigue and dissatisfaction. We have therefore weakened statements suggesting that users do not reject AIGC itself or that negative appraisals are the only relevant mechanisms. We also clarified that positive and neutral evaluations of AIGC were not measured, and that the present model cannot determine whether AI origin itself affects user responses independently of evaluative valence.

Comment 21

Response: Thank you for this clarification. We agree that a larger standardized coefficient does not by itself demonstrate a statistically significant difference between coefficients. Because no formal Wald or bootstrap coefficient-comparison test was conducted, we have removed the wording “strongest predictor” and “strongest mechanism.” We now describe perceived low-quality prevalence as having the largest estimated standardized coefficient among the three appraisal paths, without claiming that it was statistically stronger than the other predictors.

Comment 22

Response: Thank you for this important clarification. We agree that the original practical recommendations extended beyond the interventions tested in this study. We have revised the Practical implications section to present granular AIGC labels, recommendation controls, automated duplicate detection, quality-sensitive moderation, and prioritization of human-created content as tentative design proposals for future research. We now explicitly state that their effectiveness was not tested in the present study.

Comment 23

Response: Thank you for recommending these recent studies. We have considered and cited both articles in the revised Discussion.

The recent AI-fatigue study provides a useful conceptual and measurement benchmark by treating AI fatigue as a multidimensional construct involving cognitive, emotional, behavioral, and physical components [46]. We now cite this work to situate the present AIF construct, while clarifying that our study focuses specifically on cognitive and affective fatigue associated with AI-generated short-video content.

We also cited the recent study on critical thinking in generative AI use, which emphasizes verification, epistemic motivation, and reflective judgment as dimensions of critical engagement with AI outputs [47]. This work supports our discussion of the cognitive effort involved in identifying, filtering, and evaluating AIGC.

Because these studies were published after our data collection and were not part of the original questionnaire, we did not retroactively adopt their scales or alter the current analyses. Instead, we present them as recent conceptual and measurement references and as directions for future research.

Comment 24

Response: Thank you for this important comment. We agree that the original wording overinterpreted the weak result for perceived narrative homogenization. Because the bootstrap confidence interval included zero, we no longer describe this finding as “theoretically meaningful” or “marginally robust.” We now report the result as statistically unsupported and acknowledge that the estimate may be sensitive to measurement overlap among appraisal constructs, shared-variance suppression or redistribution, and sampling uncertainty.

Reviewer 2,

Comment 1

Response: Thank you for this helpful suggestion. We agree that the previous version repeated the definitions of the three appraisal constructs across several sections. We have reorganized the manuscript to clarify the progression from concepts to hypotheses and measurement. The Introduction now provides only concise definitions of PLQ, PNH, and PCO. The hypothesis-development section focuses on their theoretical roles within Cognitive Appraisal Theory and the S–O–R framework. The Measures section now reports only item numbers, referent period, item sources, response format, and coding information. Repetitive construct definitions have been removed from the Discussion and Conclusion.

Comment 2

Response: Thank you for this important comment. We agree that the development of the AIGC-specific measures required a more transparent description. We have now clarified the item-generation process, theoretical basis, expert review, item revision and deletion decisions, absence of participant pilot testing, and final item-retention procedure.

PAS, PLQ, and PNH were newly developed from their operational definitions and the characteristics of AI-generated short-video experiences. PCO items were adapted from the information-overload dimension of the Technology Overload Scale [34]. AIF items were adapted from the cognitive and emotional dimensions of the Social Media Fatigue Scale [13]. SAT and CVI items were adapted from satisfaction and continuance-intention measures based on the expectation-confirmation model [35].

Before formal data collection, five experts independently reviewed the candidate items in one review round. Their backgrounds covered media psychology, communication and human–computer interaction, psychometrics and statistics, survey methodology, and short-video industry or content governance. The review focused on relevance, clarity, comprehensibility for Chinese short-video users, and contextual appropriateness.

Based on the consolidated written feedback, several items were revised to clarify construct boundaries and avoid ambiguous or potentially causal wording. PLQ8 was removed before formal administration because it assessed perceived informational value rather than the audiovisual, logical, and production-quality characteristics defined by PLQ. The final questionnaire retained PLQ1–PLQ7. No separate participant pilot study or formal cognitive interview was conducted. The complete item-source, revision, deletion, and retention information is provided in Supplementary Table S1.

Comment 3

Response: Thank you for identifying this inconsistency. PLQ8 was an initial candidate item in the questionnaire-development stage but was removed after expert content review and before formal data collection. The item assessed perceived informational value rather than the audiovisual, logical, and production-quality characteristics defined by PLQ. Therefore, the deletion was theory- and content-based rather than data-driven.

PLQ8 was not included in the final administered questionnaire, the analytic dataset, or any statistical analysis. The final PLQ measure therefore consisted of PLQ1–PLQ7. Because PLQ8 was removed before data collection and no responses to this item were available, no statistical robustness analysis involving PLQ8 was applicable. The deletion decision was made before inspecting formal survey responses and is now clarified in the Methods and Supplementary Table S1.

Comment 4

Response: Thank you for raising this important concern. We agree that some PCO and AIF items refer to related experiences involving attention and information processing. We have clarified their conceptual distinction: PCO represents a resource-oriented appraisal of the perceived demands involved in identifying, filtering, and evaluating AI-generated videos, whereas AIF represents a subsequent fatigue state involving cognitive exhaustion, irritation, boredom, and reduced viewing interest.

Empirically, the latent correlation between PCO and AIF was .612, and their HTMT value was .608. In addition, the two-factor model fit substantially better than the one-factor model, with Δχ²(6) = 518.186, p < .001, and ΔCFI = .057. These results support their empirical distinction. However, we acknowledge that semantic similarity in references to attention and processing may have increased their observed association. We have added this possibility to the Limitations section and now interpret the discriminant-validity evidence cautiously.

We again thank the Academic Editor and the two reviewers for their time and constructive guidance. We appreciate the opportunity to revise the manuscript and hope that the revised version is now suitable for further consideration.

Sincerely,

The Authors

Attachment Submitted filename: Response to Reviewers.docx

# Peer review: Decision Letter 1

**DOI:** 10.1371/journal.pone.0360731.r003

**Authors:** Donghwa Chung

30 Sep 2026

<p>Perceived AIGC saturation and AI content fatigue on short-video platforms: An appraisal-based structural equation modeling study

PONE-D-26-35682R1

Dear Dr. Tang,

We’re pleased to inform you that your manuscript has been judged scientifically suitable for publication and will be formally accepted for publication once it meets all outstanding technical requirements.

Within one week, you’ll receive an e-mail detailing the required amendments. When these have been addressed, you’ll receive a formal acceptance letter and your manuscript will be scheduled for publication.

An invoice will be generated when your article is formally accepted. Please note, if your institution has a publishing partnership with PLOS and your article meets the relevant criteria, all or part of your publication costs will be covered. Please make sure your user information is up-to-date by logging into Editorial Manager at Editorial Manager® and clicking the ‘Update My Information' link at the top of the page. For questions related to billing, please contact billing support.

If your institution or institutions have a press office, please notify them about your upcoming paper to help maximize its impact. If they’ll be preparing press materials, please inform our press team as soon as possible -- no later than 48 hours after receiving the formal acceptance. Your manuscript will remain under strict press embargo until 2 pm Eastern Time on the date of publication. For more information, please contact onepress@plos.org.

Kind regards,

Donghwa Chung

Academic Editor

PLOS One

Additional Editor Comments (optional):

**I sincerely appreciate the reviewers’ time and effort in reviewing the revised manuscript. The reviewers provided critical and constructive comments regarding the theoretical, analytical, and other aspects of the manuscript, which helped identify issues in the previous version.**  In the current version, the authors have made substantial revisions and adequately addressed all reviewers’ comments point by point.

Please note that there are a few minor formatting issues throughout the manuscript. Please check the font consistency throughout the manuscript. Some lines in Table 4 are missing, so please keep the same table style as Tables 1–3. In addition, some paragraphs are left-aligned while others are center-aligned. Please ensure consistent alignment throughout the manuscript.

These are only minor formatting issues. Other than that, I have no further suggestions and recommend acceptance of the manuscript in its current form.

Reviewers' comments:

Reviewer's Responses to Questions

**Comments to the Author**

Reviewer #1: All comments have been addressed

Reviewer #2: All comments have been addressed

**********

2. Is the manuscript technically sound, and do the data support the conclusions?

Reviewer #1: Yes

Reviewer #2: Yes

**********

3. Has the statistical analysis been performed appropriately and rigorously?

Reviewer #1: Yes

Reviewer #2: Yes

**********

4. Have the authors made all data underlying the findings in their manuscript fully available?

The PLOS Data policy

Reviewer #1: Yes

Reviewer #2: Yes

**********

5. Is the manuscript presented in an intelligible fashion and written in standard English?

Reviewer #1: Yes

Reviewer #2: Yes

**********

Reviewer #1: The revised manuscript has addressed my substantive concerns well. The measurement development, analytical reporting, discriminant-validity evidence, control analyses, and interpretation of cross-sectional findings are now transparent and appropriately cautious.

Reviewer #2: The authors have responded carefully and comprehensively to the previous comments. The revised manuscript substantially improves transparency. The limitations of the measurement strategy and AIGC identification are also now explicitly acknowledged. I therefore recommend acceptance.

**********

what does this mean?). If published, this will include your full peer review and any attached files.

If you choose “no”, your identity will remain anonymous but your review may still be made public.

**Do you want your identity to be public for this peer review?**  For information about this choice, including consent withdrawal, please see our Privacy Policy

Reviewer #1: No

Reviewer #2: No

**********

# Editor report: Acceptance letter

**DOI:** 10.1371/journal.pone.0360731.r004

**Authors:** Donghwa Chung

PONE-D-26-35682R1

PLOS One

Dear Dr. Tang,

I'm pleased to inform you that your manuscript has been deemed suitable for publication in PLOS One. Congratulations! Your manuscript is now being handed over to our production team.

At this stage, our production department will prepare your paper for publication. This includes ensuring the following:

* All references, tables, and figures are properly cited

* All relevant supporting information is included in the manuscript submission,

* There are no issues that prevent the paper from being properly typeset

You will receive further instructions from the production team, including instructions on how to review your proof when it is ready. Please keep in mind that we are working through a large volume of accepted articles, so please give us a few days to review your paper and let you know the next and final steps.

Lastly, if your institution or institutions have a press office, please let them know about your upcoming paper now to help maximize its impact. If they'll be preparing press materials, please inform our press team within the next 48 hours. Your manuscript will remain under strict press embargo until 2 pm Eastern Time on the date of publication. For more information, please contact onepress@plos.org.

You will receive an invoice from PLOS for your publication fee after your manuscript has reached the completed accept phase. If you receive an email requesting payment before acceptance or for any other service, this may be a phishing scheme. Learn how to identify phishing emails and protect your accounts at https://explore.plos.org/phishing.

If we can help with anything else, please email us at customercare@plos.org.

Thank you for submitting your work to PLOS One and supporting open access.

Kind regards,

PLOS One Editorial Office Staff

on behalf of

Dr. Donghwa Chung

Academic Editor

PLOS One
